# Epigenetics of amphetamine-induced sensitization: HDAC5 expression and microRNA in neural remodeling

**DOI:** 10.1186/s12929-016-0294-8

**Published:** 2016-12-08

**Authors:** Philip K. Liu, Christina H. Liu

**Affiliations:** Department of Radiology, Molecular Contrast-Enhanced MRI Laboratory at the Athinoula A. Martinos Center for Biomedical Imaging, Massachusetts General Hospital and the Harvard Medical School, CNY149 (2301) Thirteenth Street, Charlestown, MA 02129 USA

**Keywords:** Amphetamine, Brain repair, Compartmentalization of iron oxide, MCE MRI, Drugs of abuse, Microglia, miRNA, Molecular imaging, Nanoparticles, Endogenous progenitors, Precision delivery, Signal sensitivity, Target specificity

## Abstract

**Background:**

Histone deacetylase (HDAC) activities modify chromatin structure and play a role in learning and memory during developmental processes. Studies of adult mice suggest HDACs are involved in neural network remodeling in brain repair, but its function in drug addiction is less understood. We aimed to examine in vivo HDAC5 expression in a preclinical model of amphetamine-induced sensitization (AIS) of behavior. We generated specific contrast agents to measure HDAC5 levels by in vivo molecular contrast-enhanced (MCE) magnetic resonance imaging (MRI) in amphetamine-naïve mice as well as in mice with AIS. To validate the MRI results we used ex vivo methods including in situ hybridization, RT-PCR, immunohistochemistry, and transmision electron microscopy.

**Methods:**

We compared the expression of HDAC5 mRNA in an acute exposure paradigm (in which animals experienced a single drug exposure [A1]) and in a chronic-abstinence-challenge paradigm (in which animals were exposed to the drug once every other day for seven doses, then underwent 2 weeks of abstinence followed by a challenge dose [A7WA]). Control groups for each of these exposure paradigms were given saline. To delineate how HDAC5 expression was related to AIS, we compared the expression of HDAC5 mRNA at sequences where no known microRNA (miR) binds (hdac5AS2) and at sequences where miR-2861 is known to bind (miD2861). We synthesized and labeled phosphorothioated oligonucleic acids (sODN) of hdac5AS2 or miD2861 linked to superparamagentic iron oxide nanoparticles (SPION), and generated HDAC5-specific contrast agents (30 ± 20 nm, diameter) for MCE MRI; the same sequences were used for primers for TaqMan® analysis (RT-qPCR) in ex vivo validation. In addition, we used subtraction R2* maps to identify regional HDAC5 expression.

**Results:**

Naïve C57black6 mice that experience acute exposure to amphetamine (4 mg/kg, by injection intraperitoneally) show expression of both total and phosphorylated (S259) HDAC5 antigens in GFAP^+^ and GFAP^−^ cells, but the appearance of these cells was attenuated in the chronic paradigm. We found that MCE MRI reports HDAC5 mRNA with precision in physiological conditions because the HDAC5 mRNA copy number reported by TaqMan analysis was positively correlated (with a linear coefficient of 1.0) to the ΔR2* values (the frequency of signal reduction above background, 1/s) measured by MRI. We observed SPION-mid2861 as electron dense nanoparticles (EDNs) of less than 30 nm in the nucleus of the neurons, macrophages, and microglia, but not in glia and endothelia. We found no preferential distribution in any particular type of neural cells, but observed scattered EDNs of 60–150 nm (dia) in lysosomes. In the acute paradigm, mice pretreated with miD2861 (1.2 mmol/kg, i.p./icv) exhibited AIS similar to that exibited by mice in the chronic exposure group, which exhibited null response to mid2861 pretreatment. Moreover, SPION-miD2861 identified enhanced HDAC5 expression in the lateral septum and the striatum after amphetamine, where we found neurprogenitor cells coexpressing NeuN and GFAP.

**Conclusions:**

We conclude that miD2681 targets HDAC5 mRNA with precision similar to that of RT-PCR. Our MCE MRI detects RNA-bound nanoparticles (NPs) in vivo, and ex vivo validation methods confirm that EDNs do not accumulate in any particular cell type. As HDAC5 expression may help nullify AIS and identify progenitor cells, the precise delivery of miD2861 may serve as a vehicle for monitoring network remodeling with target specificity and signal sensitivity after drug exposure that identifies brain repair processes in adult animals.

**Electronic supplementary material:**

The online version of this article (doi:10.1186/s12929-016-0294-8) contains supplementary material, which is available to authorized users.

## Background

Amphetamine-type stimulants are associated with aggressive behavior as well as behaviors like those characteristically seen in schizophrenia and bipolar syndrome in humans [1]. One of the pathways that rewards these behavioral manifestations involves the brain’s ventral tegmental area (VTA), the origin of the dopaminergic cell bodies of the mesocorticolimbic dopaminergic pathway [2]. GABAergic neurons in the VTA send projections to brain regions including the nucleus accumbens (NAc), striatum (Cpu), and pre-frontal cortex (PFC) in the forebrain, and to the hippocampus through the lateral septum (LS). The LS lies below the rostrum of the corpus callosum; neurons of the LS receive reciprocal connections from the olfactory bulb, hippocampus, amygdala, hypothalamus, midbrain, habenula, cingulate gyrus and thalamus [3]. The LS is considered a pleasure/fear zone in animals, and plays a role in reward and reinforcement along with the NAc [4–6]. Epigenetic regulatory events have been shown to mediate the lasting effects of drugs of abuse; one possible means of such epigenetic regulation is chromatin remodeling via histone modifications. Acetylation by histone acelytransferases (HATs) promotes transcriptional activity by relaxing chromatin through the insertion of an acetyl group to the lysine residue of histone. However, HDACs catalyze deacetylation of acetylated chromatin, removing the modification by HATs in chromatin. One of the class IIa HDACs that have cell-type-restricted patterns of expression, HDAC5 associates with HDAC3 in vivo to shuttle between the nucleus and cytoplasm [7, 8]. HDAC5 is known to be involved in learning and memory function, as well as playing a role in axon regeneration in repair [9–11]. Other reports have suggested, as observed in various models of addictive drugs, that reduced HDAC activity may be involved in neural plasticity [12–14]. However, the mechanism by which HDAC5 may act in neural repair in adults remains unclear.

Recent studies have suggested that HDAC5 activity may be regulated by miR-2861 [15, 16]. Endogenous miRNAs are noncoding RNAs that bind to specific gene transcripts, modulate gene expression and play key roles in the regulation of developmental processes [17]. The possible associations between miR-2861 and HDAC5 expression prompted us to investigate the role of endogenous miR-2861 and HDAC5 in the context of psychostimulant sensitization.

Our goal is to investigate how AIS is associated with endogenous miR-2861 and HDAC5 expression after chronic amphetamine exposure. We have developed NPs that specifically target two segments of HDAC5 mRNA: miD2861 and hdac5AS2. We conjugated SPION (core diameter 5–10 nm, hydrodynamic diameter 30 ± 20 nm) via Schiff-base reduction with biotinylated miD2861 or hdac5AS2 [18]. Upon binding to gene transcripts, these NPs produced target-specific signal reduction in T2*-weighted MR images [19–21], which we converted to R2* (1/s) for quantitation. As R2* values above background (ΔR2*) are positively proportional to iron concentration [22], our data demonstrate that together SPION-miD2861 and MCE MRI measure HDAC5 mRNA in vivo with accuracy similar to that of reverse transcription PCR. Using noninvasive MRI in a preclinical mouse model of chronic amphetamine exposure, we demonstrated that SPION-miD2861 monitors regional HDAC5 expression in the LS, where we found endogenous progenitor cells. These results suggest miD2861 may monitor neural repair and remodeling.

## Methods

### Animals and housing

All of the procedures used in this study were approved by the Massachusetts General Hospital Subcommittee on Research Animal Care, the institutional animal welfare committee, in accordance with the Public Health Service Guide for the Care and Use of Laboratory Animals. Adult male C57black6 mice (Taconic Farm, Germantown, NY) (*n* ≥ 3 litters at a time), 2 to 3 months of age and weighing 23 ± 2 g, or transgenic mice expressing green fluorescent protein (GFP) directed by Fos promoter [B5;DBA-Tg(Fos-tPA, fos-EGFP*)1Mmay Tg(terO-lacZ, tPA*)1Mmay/J] (Jackson Lab, ME) were kept in cages with sawdust bedding, in a room with controlled light cycles (12 h light/12 h dark). All animals had free access to water, and were fed standard lab chow. Mice were trained, operated on, and tested in a random manner; a blinded observer performed the behavioral testing. All contrast agents and aptamers were delivered using BBB bypass to mice with icv puncture [23, 24], through which the distribution of agents has been shown to be more uniform than by icv alone.

**Fig. 1:**
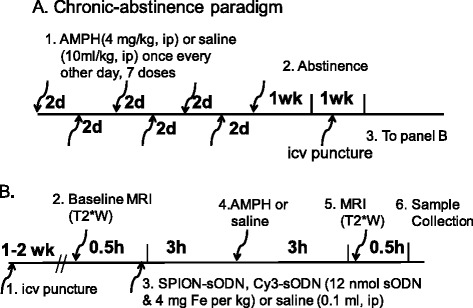
Panel **a** Summary of amphetamine sensitization using chronic exposures to amphetamine or saline with abstinence. Panel **b** Summary of protocol for MRI acquisition and amphetamine (acute or challenge) administrations

For each series of MRI experiments, we started with eight mice (*n* = 4 each for acute and chronic paradigms, Fig. 1). Mice in each paradigm received amphetamine or saline (*n* = 2 each); we repeated the series of experiments until we had gathered data on the appropriate number of animals, as determined by power analysis. We used a double-blind design for all experiments, in which sample preparation and data acquisition were blinded, and the samples were given coded identifiers. Following MRI and photography the coded datasets were delivered to and decoded by the Principal Investigator.

### Amphetamine exposure paradigms

For the chronic exposure paradigm, eight age-matched, amphetamine-naïve, male C57black6 mice received a single dose of amphetamine in their home cage every other day, for a total of seven injections of amphetamine (4 mg/kg, by injection intraperitoneally [i.p.] A7) [18, 25–28]; this was followed by 2 weeks with no drug exposure (abstinence, A7W in Fig. 1a). A final dose of amphetamine or saline was given on the day of post-SPION MRI, such that we could compare the effect of a challenge dose of amphetamine following chronic exposure and abstinence in our A7WA group to control groups without challenge dose (A7WS or S7WS). For the acute exposure paradigm (A1), age-matched, amphetamine-naïve, male C57black6 mice received a single dose of amphetamine (4 mg/kg, i.p.); the control group received a single dose of saline (S1, vehicle, 10 ml/kg, i.p.).

### Immunohistochemistry of total and phosphorylated HDAC5 antigens in amphetamine exposure paradigms

For ex vivo assays we examined two C57black mice in the A7W group, as well as four normal naïve mice with saline or acute amphetamine exposure (*n* = 4 each); these six mice received no icv puncture, nor were they given contrast agent. We administered saline (100 μl, i.p.) or amphetamine (4 mg per kg, i.p.) 3 h before the mice (*n* ≥ 2, each group) were put under general anesthesia and retrograde-perfused with ice-cold saline. Isolating brain tissue as described previously [21], we stained slices of brain tissue (25 μm in thickness) with total or phosphorylated HDAC5 antigens with ab1439 (Abcam) and ab192339 (phosphor S259, detects HDAC5 phosphorylated at Serine 259), respectively. We then co-stained Cy2-labeled rabbit polyclonal IgG against glial fibrillary acidic protein (GFAP, Z0334, Dako) or Cy3-labeled polyclonal IgG against ionized calcium-binding adaptor molecule 1 (IBA1, ab5076, Abcam); we used Cy2-labeled monoclonal IgG against NeuN (A60, Millipore) or Cy3-labeled goat IgG against GFAP (clone C-19, Santa Cruz) for progenitor cells. Nucleic acids were stained with DAPI (1:500 dilution) [18]. To examine HDAC5 mRNA expression using Cy3-sODN (see below), transgenic mice (*n* = 3) underwent icv puncture 1 week before they were administered Cy3-sODN (120 pmol in 0.1 ml, i.p.) and a dose of amphetamine, as in the acute paradigm (Fig. 1b). All histological images were acquired using the same exposure time and gain, using a Retiga EXi camera on an Olympus microscope and cellSens Dimension software (Olympus America Corp, Nashua, NH).

### Biotinylated sODN for HDAC5 transcripts

We designed two sODNs with antisense sequence to HDAC5 mRNA; for all sODNs we used nucleotide BLAST to validate target mRNA with potential binding (http://blast.ncbi.nlm.nih.gov/Blast.cgi). The sequences were obtained from GenBank (AF207748): sODN of HDAC5 mRNA or miR-2861 binding site (hdac5) = 5′-aggctgagaggcaggccctt-3′ (forward to 1188–1169); miD2861 = 5′ aagggcctgcctctcagcct-3′ (antisense to 1188–1169) and its upstream primer (USP)1 = 5′-acggctttactggctcagtc-3′ (forward to 971–980); HDAC5AS2 = 5′-atctcattccacaccgtgtc-3′ (antisense to 2341–2360) and its USP2 = 5′- tcaaggatgaggatggcgag-3′ (forward to 1781–1800); passenger of matured miR-2861 = cuccggcucccccuggccucccgg (passenger); hairpin and miR-2861 = gaacuacaagucccagggggccuggcggcgggcggag (premiR-2861); antisense to premir-2861 = ccctgggacttgtagttc (mpremiR-2861a). All primers were phosphorothioated by replacing non-bridging oxygen with sulfur on the phosphate linkages. We attached one biotin to the 3′ termini. We also synthesized sODNs for pre-miR-2861 and matured miR-2861 [15]. We stored all of these sODNs in aliquots of 0.05 ml (0.1 μM), at −20 °C.

### Target binding and cellular retention

We mixed Cy3-miD2861 with one sODN of four different sequences (the passenger of miR-2861, matured miR-2861, Cy3-miD2861 and cDNA of HDAC5 mRNA at the miR-2861 binding site) at a 1:1 ratio (100 pmol each in 50 μl) at room temperature. For ex vivo hybridization, we heated the mixture at 65 °C for 5 min, then slowly cooled it on a thermocycler (at a rate of 1° drop per minute) to 20 °C, where it was maintained for 30 min. We resolved the hybrids in agarose gel (1.5%) using gel electrophoresis, and obtained photographs at 495 nm/521 nm (excitation and emission spectrum peak wavelengths) using an Imager FluorChem Q (Alpha Innotech, CA). To test binding ability in vivo, we transfected Cy2-miD2861 (30 pmol) to PC-12 cells in a 35 mm (dia) culture dish on a microscope stage incubator chamber with humidified air comtaining 5% CO2, at 37 °C (model INU-UK-F1; Tokai Hit, Shizuoka, Japan) as described [29]. We washed and changed DNA-free medium 3 h later, then transfected Cy3-sODNhdac5 to both plates. We acquired live cell images at 18 and 42 min and 2 h after Cy3-sODNhdac5 using automatic time-lapse photography with constant exposure time and image gain (CellSense Imaging Software, Olumpus). Representative cell images were processed using Adobe Photoshop CS2 (Adobe Systems, San Jose, CA).

### Modular contrast agent using SPION-NA

We synthesized NeutrAvidin (NA)-labeled Molday Ion (CL-30Q02-2, BIOPAL, Worcester MA) using the protocol previously published [20]. This commercially available Molday Ion contains 6 k–9 k molecules of iron oxide (dextran-coated superparamagnetic iron oxide nanoparticles, or SPION): it has a unique Zeta potential (−5 mV) with an effective size of 25 nm (dia) and relaxivities of R1 = 15.4 and R2 = 33.9 s^−1^mM-^1^. Briefly. we conjugated SPION-NA with biotinylated aptamer (3 nmol biotinylated sODN per mg SPION-NA) by mixing. The resulting SPION-sODN remained at 4 °C for 16 h before it was administered to animals. Before delivery we added 1 μg of lipofectamine (Lipofectamine 2000, Invitrogen) to the mix.

### Intracerebroventricular (icv) puncture to create a BBB-bypass port

One week before SPION-sODN or sODN delivery we anesthetized the mice with pure O_2_ plus 2% halothane at a flow rate of 800 ml/min. We performed icv puncture (LR −1; AP −0.4 and DV: −3 mm, bregma) using a 28G needle to create a BBB-bypass port, and afterward sealed the skull with bone wax, then sutured and disinfected the wound (Povidone-Iodine, Medline Ind, Mundelein, IL). The BBB remained open for approximately 21 days after the icv procedure; we utilized this 21-day window to deliver NPs or sODN by injection intraperitoneally (i.p.) [23, 24]. On the day of MRI, we acquired baseline MRI in a group of four mice (30 min each). We eliminated any mice that exhibited baseline R2* values more than one standard deviation of the average R2* values in normal brains (*n* >500) we had examined in previous studies (stratification). However, we note that the need to eliminate animals based on this criterion is rare.

### Dynamics of SPION-sODN uptake

Before SPION delivery, we acquired baseline T2*-weighted (T2*W) MRI scans (30 min each scan) on four mice identified individually as mice A, B, C, and D, and immediately afterward delivered SPION-hdac5AS2 or SPION-miD2861 (4 mg Fe or 12 nmol sODN per kg, i.p.) (Fig. 1b). Each mouse remained in awake in its home cage. We acquired MRI at 2-h intervals following SPION-sODN from mice A to D, discontinuing MRI acquisition at 6 h. We repeated the evaluation until we had gathered data from enough mice (*n* = 4 at each time point, or as determined by power analysis).

### Cy3-sODN-hdac5AS2 uptake using in vivo hybridization

We examined the distribution of Cy3-sODN-hdac5AS2 using in vivo hybridization and ex vivo assay [23]. We delivered Cy3-sODN-hdac5AS2 (12 nmol per kg, i.p) to transgenic mice with icv port, and 3 h later administered amphetamine (4 mg per kg, i.p.). Four hours after amphetamine administration we obtained brain samples and froze them by slow cooling with liquid nitrogen. After staining slices of brain tissue (25 μm in thickness) with 0.5% Hoechst for nucleic acid, we obtained photographs using the same technique and equipment described above [18].

### Molecular contrast-enhanced (MCE) MRI in vivo

We acquired background T2*W MRI and delivered SPION-hdac5AS2 or SPION-miD2861 to four mice, as shown in Fig. 1b; 3 h later we injected amphetamine (4 mg per kg) or saline (100 μl, i.p.). We acquired MRI 3 h after amphetamine, and statistically analyzed SPION retention in various regions of interest (ROIs). We used the mean and SD from the first pair (*n* = 2) in each paradigm to compute the sample size needed to avoid type II error for SPION-actin uptake at each time point. This MRI acquisition was repeated in enough mice to achieve adequate sample size, according to power analysis (see Statistical analysis below).

We performed MCE MRI using a 9.4 T MRI system (Bruker Avance system, Bruker Biospin MRI, Inc., Billerica, MA). We measured R2* changes before and after NP delivery, and evaluated SPION-labeled gene expression in the R2* maps acquired using multi-echo gradient echo sequences; TR 800 ms, six echoes (TE = 1.94, 3.41, 4.88, 6.35, 7.82, 9.29 ms) with spatial resolution of 0.1 mm × 0.1 mm × 0.25 mm. We followed the same protocol described in our previous publications [21] for stratification before MCE MRI, data acquisition, data analysis and examinations of within- and between-litter differences.

### MCE MRI data analysis

We have used subtraction R2* maps of the chronic paradigm and acute paradigm and found ROI with neurogliosis based on elevated GFAP mRNA expression [29]. Because R2* values above baseline are positively proportional to iron concentration [22], where R2* is the rate of signal reduction (R2* = 1/T2*, s^−1^), we compared the R2* maps from all T2*W MRI scans in the regions contralateral to the hemisphere with icv port. We aligned T2*W MRI using the “jip analysis toolkit” (URL: http://www.nmr.mgh.harvard.edu/~jbm/jip/). Any R2* values greater than three standard error of the mean (SEM) of the average pre-SPION R2* values were considered significantly different from the background. We computed the ΔR2* as the increase in R2*_SPION-sODN brain_ above the background: i.e., R2* post SPION-sODN - (R2*_baseline_ + R2*_3 SEM of baseline_), or as a percent elevation above the baseline (ΔR2*/average baseline R2*) ×100%. Any R2* values above the baseline R2* were shown in the color scale.

### Contrast-to-noise ratio (CNR)

The noise in the ROI comes from the background before contrast agent delivery. CNR is defined as the ratio of the difference between two image signals to the square root of the standard deviation of the background noise. For our purposes, baseline R2* maps showing endogenous iron levels serve as ‘background,’ and their standard deviations are the ‘noise’ to R2* maps of brains containing SPION-sODN. Therefore, we defined the CNR representative of SPION-sODN uptake in each ROI, and at any given time point, as the change in contrast, i.e., ΔR2* divided by noise (the square root of the standard deviation of R2* within the same ROI in baseline brains).

### Validation of SPION-sODN delivery using transmission electron microscopy (TEM)

We collected tissue samples immediately after MRI; the left NAc of S1 and A1 mice was immersed in 2.5% PBS-buffered glutaldehyde at 4 °C and sent to the TEM laboratory of the Histology Core Facility at the MGH Center for Systems Biology for preparation and double-blinded examination. After tissue was dehydrated in ascending concentrations of ethanol, immersed in propylene oxide, and embedded in Epon 812 resin (Agar Scientific Ltd., Standstead, England), samples were cut into ultrathin sections (~60 nm). The Core prepared tissue with and without standard TEM stain using osmium tetroxide (1%, 2 h), uranyl acetate (Ua, 2%, 5 min) and Reynold’s lead citrate [20]. We found that standard staining masked NP identification; we modified TEM staining by omitting all stains, unless indicated, to reduce the background of membrane structure and enable visualization of SPION. The neuronal nucleus was identified as a smooth, round nuclear body with diameter of ~7 μm. We defined microglia (MG) by the presence of irregular euchromatic nucleus, a peripheral rim of heterochromatin, and various empty and partially filled lysosomes/exosomes (Ly/Ex).

### Ex vivo RT-qPCR methods

For TaqMan® analysis we extracted the total RNA from the brain tissue of each mouse using the RNeasy Lipid Tissue Mini Kit (Qiagen), which supplied all required buffers. For RT-qPCR, we obtained total RNA from striatal or hippocampal tissue from three groups of mice that were administered saline (S), or acute (A1) or chronic paradigms of amphetamine. The total RNA from each mouse was reverse-transcribed using oligo (dT)_25_, and the SuperScript III First-Strand Synthesis System (Invitrogen Life Technologies, Carlsbad, CA, CA). The initial RNA concentration in each sample was determined by OD260 reading and converted to the total amount of RNA. Preparation of striatal tissue from one side of each mouse brain yielded 2.8 ± 0.9 μg total RNA in 40 μl solution. From each sample, we used 280 ng, or 4 μl total RNA for cDNA synthesis in a 20 μl total volume of buffer solution; 1 μl of this solution was used for qPCR. The qPCR was performed using a TaqMan® probe-based assay (Applied Biosystems) for fosB (Assay ID: Mm00500401_m1); beta Actin (Assay ID: Mm02619580_g1) served as the internal control. We carried out relative quantification of the mRNA amount using standard SDS software, which is based on ΔΔCt models [30]. For FosB, HDAC5, and GFAP mRNA in normal brains, we measured copy number using an internal control of Actin mRNA. We did not calculate the copy number of HDAC5 mRNA in amphetamine exposure paradigms because the exposure to amphetamine might damage the brain and alter the copy number of the internal control.

### Locomotor assessments

We measured locomotor behavior according to published drug sensitization protocols [18, 25–28]. To measure horizontal locomotion and fine motor activity, we used an automated recording device (San Diego Instrument, San Diego, CA) located in the same room in which the animals were individually housed. The system has eight chambers, each of which is composed of frames equipped with five infrared photocell beams (spaced 5 cm apart) in one polypropylene cage (15 × 25 cm). The photocell beams traverse each cage in a plane above the floor. We recorded the frequency of locomotion (ambulation) as the number of sequential breaks in two adjacent beams, and measured fine motor activities (such as grooming or other stereotyped motions) by counting the number of sequential breaks in a single beam. Recordings were made every minute for at least 60 min. We reported the distance traveled as the product of 5 cm and the summation of frequencies of beam break during the time interval.

Mice were individually housed and tested in their own home cages. We pre-conditioned the mice by removing each mouse from and returning it to its cage daily for 5 days prior to behavior assessment. To examine the effect of HDAC5 knockdown, we pretreated mice with a dose of miD2861 or placebo (sODN with random sequence, or sODN-Ran) at 1.2 mmol/kg (i.p./icv) 3 h before administering amphetamine to naïve (A1) mice or mice that had been previously exposed to one dose of amphetamine (A2) or A7W), as previously described [2, 18]. We performed locomotor assessment immediately, as described above. We obtained data from twice the number of mice calculated by power analysis; the results were compared using two-way analysis of variance.

### Statistical analysis

Once we had obtained the first MRI dataset, we calculated the number of animals needed in each group to achieve at least 85% power for an *α* value of 0.05, to avoid type II error (a *post hoc* power analysis). We computed the mean and SEM from the average values in each group of animals, and compared the statistical significance of these values using a *t* test (two tail, type II or equal variant) or two-way ANOVA (GraphPad Prism IV, GraphPad Software, Inc., San Diego, CA). A *p* value of ≤ 0.05 was statistically significant [18].

## Results

**Fig. 2:**
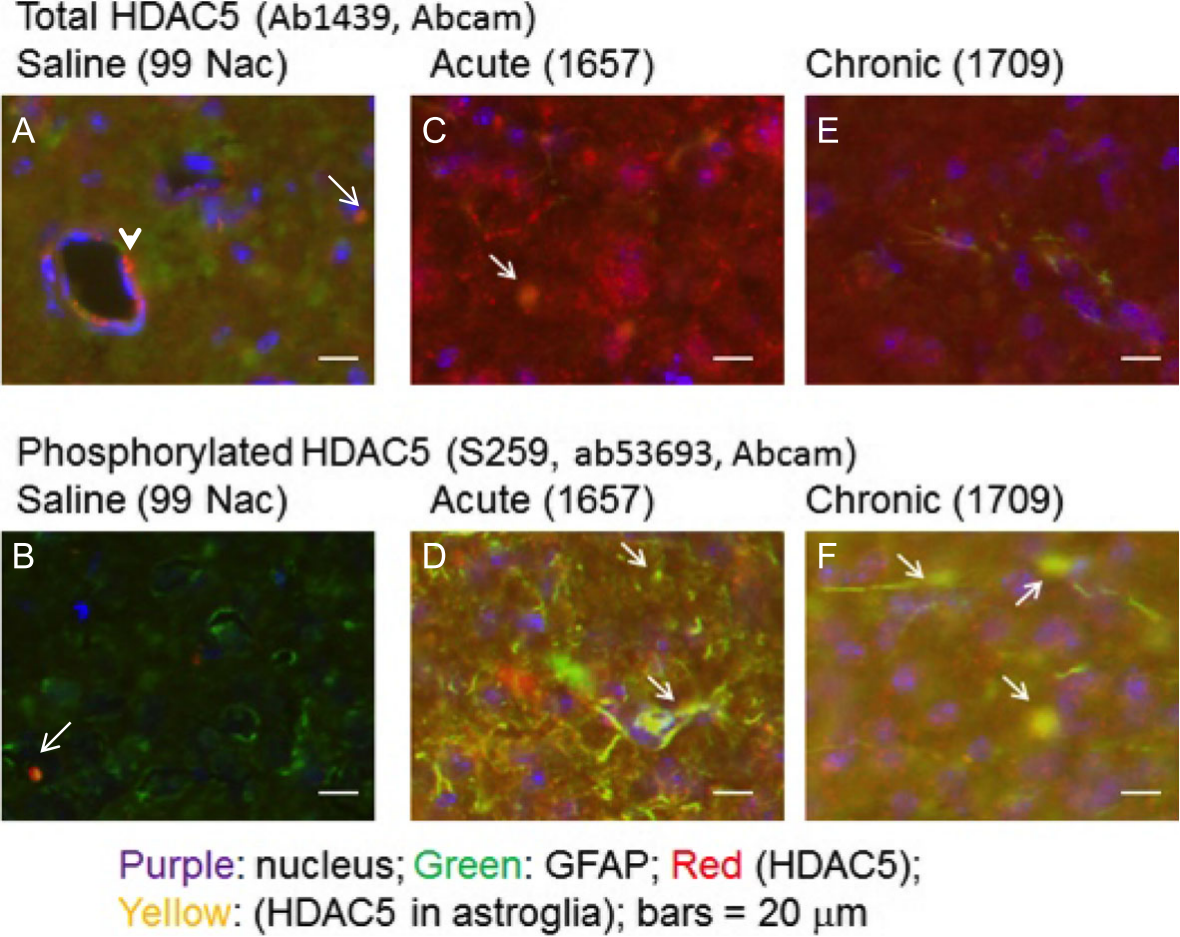
Expression of HDAC5 antigens in naïve (saline), acute and chronic exposure to amphetamine. We compared total (cy3-ab1439, Abcam) or phosphorylated HDAC5 at S259 (cy3-ab192339) in the nucleus accumbens (NAc) of mice that experienced either saline (**a** & **b**), acute amphetamine (**c** & **d**) or chronic amphetamine (**e** & **f**) exposure. Brain tissues were obtained 1 h after amphetamine or saline (i.p.) in isopropanol on dry ice. Brain slides were stained with Cy3-IgG then Cy2-gfap (Z0334, Daka), and DAPI for nucleus

We compared total and phosphorylated HDAC5 antigens [ab1439 and ab192339, respectively] in the NAc of mice that experienced either acute or chronic amphetamine exposure (Fig. 1). As Fig. 2a shows, there was some expression of total HDAC5 antigen around microvessels in saline-treated mice (GFAP^−^/HDAC5^+^, arrowhead); we observed spotty areas of HDAC5 antigen in non-astroglia (GFAP^−^/HDAC5^+^), which showed no blending yellow stains (arrows). Figure 2b shows near null expression of S259-HDAC5 antigen in the NAc without amphetamine. Detailed photographs can be found in supplemental figures (Additional file 1 and Additional file 2: Figure S1). After one exposure to amphetamine (A1) we observed both antigens of total (red, GFAP^−^/HDAC5^+^, Fig. 2c) and phosphorylated S259 HDAC5 (blending yellow, GFAP^+^/HDAC5^+^, Fig 2d, arrows) in neural cells. In addition, phosphorylated HDAC5 was visible in the soma and axons of GFAP^+^ cells (Additional file 3: Figure S2A & S2B). On the other hand, both total and phosphorylated S259 HDAC5 antigens were scarce after chronic amphetamine exposure (Fig. 2e, f; Additional file 4: Figure S3A & S3B), compared to the acute (A1) paradigm (Fig. 2, Additional file 3: Figure S2A & S2B). We observed that phosphorylated S259 antigens remained in enlarged nuclei of GFAP^+^ cells (Fig. 2f, arrows).

**Fig. 3:**
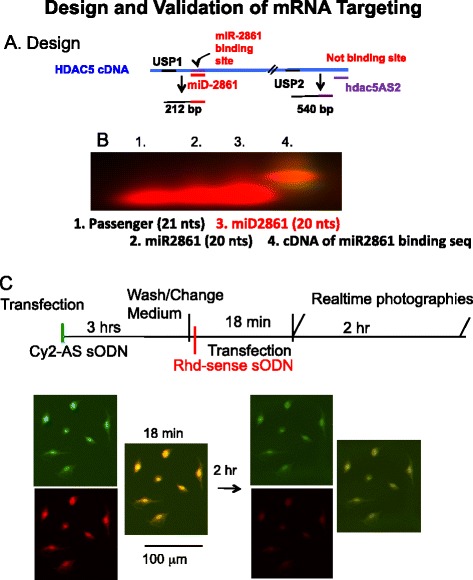
Antisense sODN binds the target sequence. Panel **a** Relative location of four primers on HDAC5 mRNA. Both miD2861 and hdac5AS2 are in AS orientation. Panel **b** Modified gel shift assay. We mixed Rhd-miD2861 with one sODN of four different primers (lane 1: the passenger of miR-2861, lane 2: matured miR-2861, lane 3: Rhd-miD2861, and lane 4: cDNA of HDAC5 mRNA at the miR-2861 binding site [sODNhdac5]). Binding of miD2861 slowed mobility during gel electrophoresis. Panel **c** Differential retention assay. We transfected Cy2-AS first for 3 h, washed, then transfected Rhd-sODN. Eighteen minutes later we washed and changed medium again, then took photography immediately and at 2 h later. Sense sODN was excluded preferentially at 2 h

**Fig. 4:**
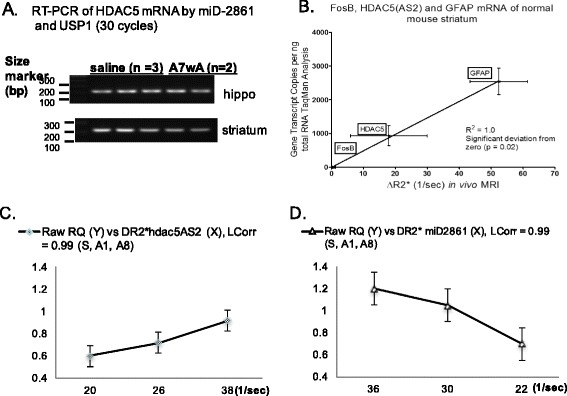
Quantitative validation of HDAC5 mRNA expression using RT-PCR. The frequencies of signal reduction above baseline (ΔR2* values) by MCE MRI positively correlate with mRNA copy numbers determined by TaqMan® analysis. Panel **a** miD2861 and upstream primer 1 (UPS1) amplified one single fragment of 212 bp of HDAC5 cDNA from brains of normal mice (saline, *n* = 3) and mice in the A7WA group (*n* = 2). Panel **b** The mRNA copy numbers of three genes and the changes in their ΔR2* values measured by MRI show positive correlation. MR data of GFAP and FosB mRNAs were from published results [18, 29]. HDAC5 data were from Fig. 5c (6 h) and Fig. 6a (S1). Panels **c** & **d** Correlations of copy numbers of HDAC5 mRNA and ΔR2* in three paradigms; MR data from Figs 6a, b and 9a., b. R^2^ or LCorr are linear correlation

We designed three additional primers (miR-2861 binding site on HDAC5 mRNA, a passenger of matured miR-2861, and premiR-2861) to demonstrate target specificity (Fig. 3a). We found that miD2861 hybridized only to the sequence of HADC5 mRNA but not to other sequences of passenger or pre-miR-2861 in an ex vivo hybridization test (Fig. 3b, Lane 4). We further demonstrated that sODNs with antisense sequences were stable in PC12 cells after transfection (Fig. 3c). Labeled sODNs with sense sequence was excluded from PC12 cells within 2 h after transfection (Fig. 3c). We then investigated precise binding of miD2861 to total cDNA that had been reverse-transcribed from HDAC5 mRNA; PCR with USP1 and miD2861 primers amplified only one fragment of 212 basepairs (bp) in samples from the hippocampus and striatum (Fig. 4a). The same was true for USP2 and hdac5AS2 (Fig. 3a) for amplification of cDNA of 580 bp (data not shown). We noted a slight variation in the intensity of the HDAC5-fragment that had been amplified from mice in the chronic paradigm. Therefore, we employed SPION-hdac5AS2 or SPION-miD2861 to measure the expression of HDAC5 mRNA in vivo.

**Fig. 5:**
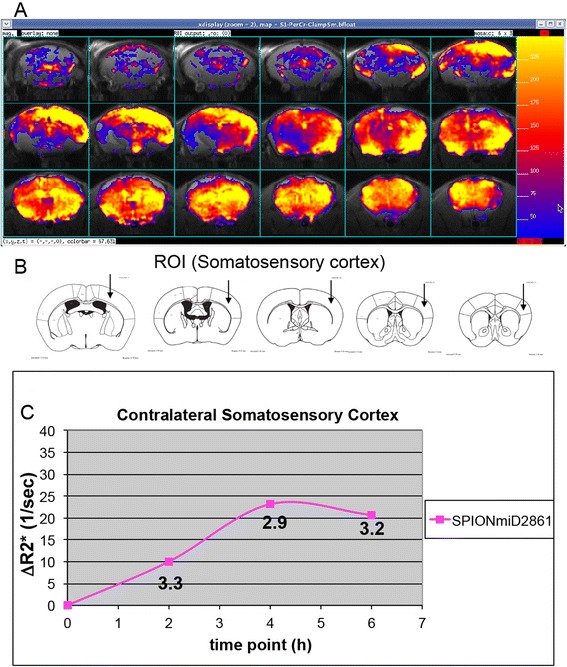
Contrast-to-noise ratio (CNR). Panel **a** Representative ΔR2* maps of 0.5 mm brain slices from the cerebellum (*upper left slice*) in percent increase from baseline (color scale was 0–250%) from one mouse (no amphetamine, SPION-miD2861). ΔR2* Maps [(Post R2*−bR2*)/bR2*) ×100%]. Panel **b** ROI (*arrows*) of the somatosensory cortex (SSC, contralateral to icv port) for data analysis [18, 20]. Panel **c** Elevations of ΔR2* values of the SSC by longitudinal MRI in vivo; mean ΔR2* values of four mice are shown. Contrast-to-noise ratio was calculated for R2* data at 2, 4, and 6 h. CNR = (R2* of mean/[SD of baseline]^1/2^)

To determine the best time to obtain optimal ΔR2* after SPION delivery, we monitored SPION-miD2861 retention in normal mouse brains (*n* = 4). Figure 5a shows R2* above baseline R2* (ΔR2*) at 4 h after delivery. We found that the average ΔR2* values in the somatosensory cortex from these mice (Fig. 5b, arrows) reached a plateau at 4 h and remained elevated at 6 h post-delivery (Fig. 5c). The CNR in the ROI had an average of 3.2 ± 0.1 from the beginning of ΔR2* elevation (at 2 h) to the peak ΔR2* elevation (at 4 & 6 h) after delivery. We found copy numbers of mRNAs from normal mouse brains correlated with the peak ΔR2* values of HDAC5, GFAP and FosB [18, 29], with a linear coefficient (r^2^) of 1.0 (Fig. 4b). The same was found for ΔR2* values using SPION-hdac5AS2 in normal mice (r^2^ = 0.97). It appears SPION-miD2861 or SPION-hadc5AS2 reported HDAC5 mRNA of normal mouse brains with the same mechanism of RT-PCR. The uptake plateau of 6 h would be used in future MRI (Fig. 1b).

**Fig. 6:**
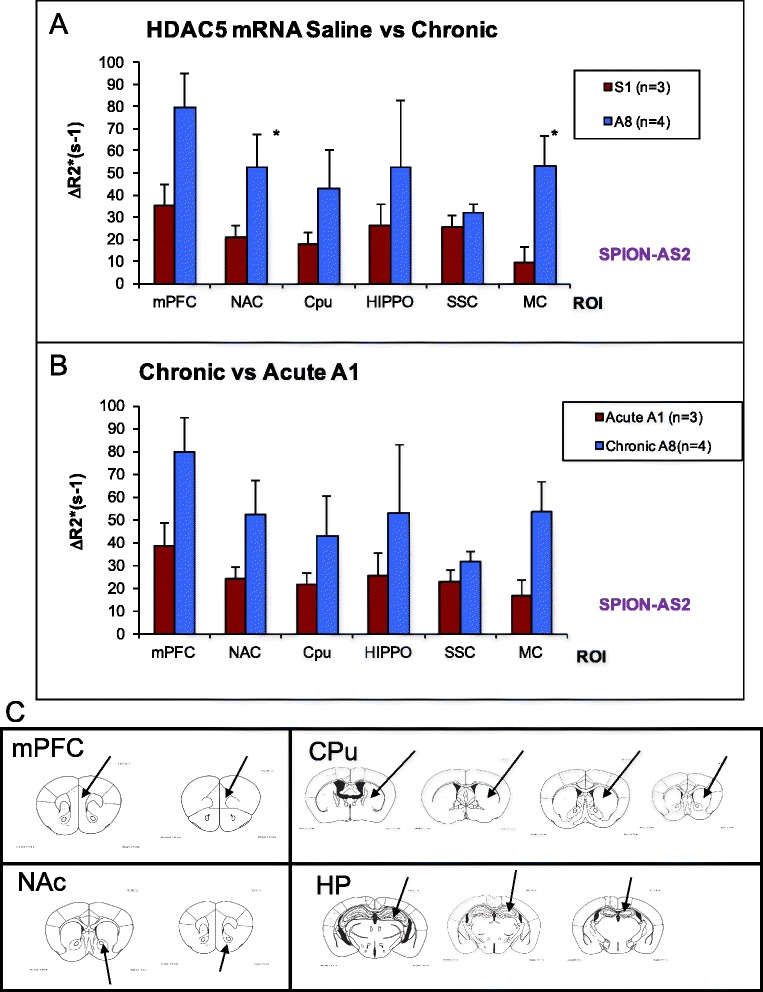
Summary of HDAC5 expression. Panels **a** & **b** Elevation of R2* values in various ROIs using SPION-hdac5AS2 and MCE MRI from mice of paradigms in Fig. 1. Panel **c** ROIs (*arrows*) of mPFC, NAc, CPu and hippocampus (HP) for data analysis

We validated the variations of HDAC5 mRNA in acute and chronic paradigms using MRI in vivo. We started with SPION-hdac5AS2, because the HADC5 sequence on the binding site of hdac5AS2 has no known interference. Figure 6 shows changes in HDAC5 mRNA expression of seven ROIs in both the acute and chronic paradigms. Compared to the control group (S1), the A1 group showed no significant differences in the medial prefrontal cortex (mPFC), NAc, and caudate putamen (CPu) of the mesolimbic pathway (S1 of Fig. 6a versus A1 of Fig. 6b). We found that animals in the chronic paradigm showed higher ΔR2* values in all ROIs compared to animals in the control and A1 groups, although these values were not significantly higher except in the NAc and motor cortex (Fig. 6a). However, the data illustrated in Fig. 6a did not support those from immunohistochemistry and RT-PCR (Fig. 2c versus 2E; 4A).

**Fig. 7:**
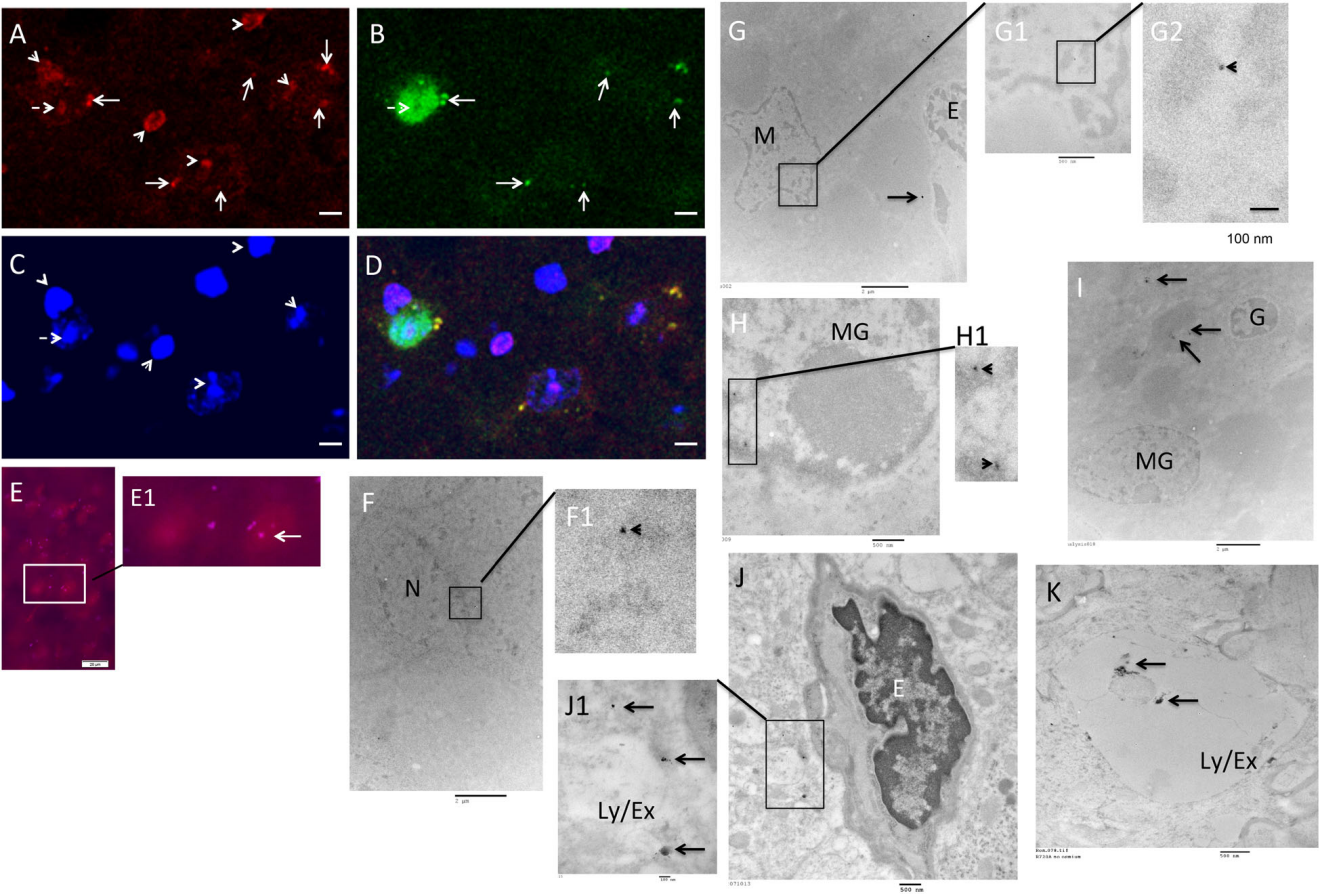
Modified in situ hybridization using Rhd-sODNhdeac5AS2 (**A**–**D**, A1) and Cy5.5-miD2861 (**E**–**J**, S1) or SPION (**K**) for HDAC5 mRNA expression. We transfected Rhd-hdac5AS2 (120 pmol) to transgenic mice expressing green fluorescent protein (GFP) directed by Fos promoter [B5;DBA-Tg(Fos-tPA, fos-EGFP*)1Mmay Tg(terO-lacZ, tPA*)1Mmay/J] according to Fig. 1b, except no MR acquisition. After we obtained brain tissues (Fig. 2), frozen slices were prepared and stained with DAPI only. Panels **A**–**C** show NAc cells with HADC5 mRNA by Rhd-sODNhdeac5AS2 (**A**), GFP (**B**) and DAPI (**C**); panel **D** shows the merged photographs of **A**–**C**. These panels show neural cells of HADC5^+^ in GFP^+^/DAPI^+^ (*broken arrows* in **A**–**C**), HDAC5^+^ in GFP^+^ (*arrows* in **A** & **B**, or *yellow* in **D**) and HADC5^+^ in GFP^−^ (*arrowheads* in **A** & **C**, *purple* in **D**). Panel **E** shows the NAc stained with goat poly-IgG against IBA1 (Ab5076, Abcam) for microglia (MG) in mice after Cy5.5-SPION-miD2861. HDAC5 mRNA is expressed in one of two MG (*arrow*, E1). Panel **F** Electron dense nanoparticles (EDN) of SPION-miD2861 were retained in the nucleus (23 nm, dia) of a neuron (N, F1 *arrowhead*). Panel **G** shows macrophage (M) and endothelium (E); we observed nuclear EDN (17 nm) in mcrophage (*arrowheads*, G2). Panel **H** shows two nuclear EDNs (28 nm each) in a microglia (H1) although other MG and glia (**G**) do not contain any EDN in the nucleus except in the lysosomes/exosomes (**I**, *arrows*). We observed several EDNs (ranging 20 to 80 nm) in Ly/Ex in close proximity of an endothelium (J1, *arrows*). Panel **K** shows EDN in the control (mice received non-targeting SPION). Bars (microns) = 20 (**E**), 6 (**A**–**D**), 2 (**F**, **G** & **I**), 0.5 (G1, **H**, **J** & **K**) or 0.1 (G2). TEM samples were without stains (**F**–**I**) or stained with uranyl acetate (2%, 5 min, **J** & **K**). See also Additional file 2: Figure S1 for our rationale for using the no stains

To investigate whether or not sODN was stable in vivo, we transfected rhd-labeled sODN-hdac5AS2 to transgenic mice expressing green fluorescent protein (GFP) directed by Fos promoter (according to the A1 paradigm) to induce cFos and hDAC5. We found rhd-sODN-hdac5AS2 was retained in the cytoplasm (GFP^+^/DAPI^−^, arrows) and nuclei (GFP^−^/DAPI^+^, arrowhead) or the nuclei of neural cells expressing GFP in cytoplasm (Fig. 7A–C, GFP^+^/DAPI^+^, broken arrows). We observed several Cy5.5-SPION-miD2861 in IBA1^+^ cells in separate experiments (Fig. 7E). We employed TEM to resolve the location of SPION-sODN retention in samples from the NAc.

Upon comparing electron dense nanoparticles (EDN) in mouse brains with TEM stain we found no significant difference in the EDNs of normal mice (Additional file 5: Figure S4A–C) compared to mice that received one optimal dose of SPION-sODN (Additional file 5: Figure S4E). Partial TEM stains (uranyl acetate and lead phosphate) showed pinocytosis of SPION-sODNs (Additional file 5: Figure S4D). We observed phagocytes with more EDN-filled Ly/Exs in mice that had been given high doses of iron (Additional file 5: Figure S4F) compared to mice given no SPION (Additional file 5: Figure S4A–C) or one optimal dose of SPION-sODN (Additional file 5: Figure S4E, G and H). The high doses we tested were 120 μg/kg (3× of optimal dose, icv delivery), or 4 mg/kg weekly (i.p) for 8 weeks in mice with large BBB opening. Although we could clearly identify EDNs of 100–500 nm (dia) in Ly/Ex in all neurons, microglia, and phagocytes of fully stained tissue, not all EDNs could be discerned; the exception was cytoplasmic EDNs of 60–150 nm in samples stained only with uranyl acetate (Additional file 5: Figure S4G1 & H1). It was only in unstained tissue that we identified EDN, of ≤ 30 nm (dia), being transported in the endoplasmic reticulum (ER) (Additional file 6: Figure S5A1), distributed in the nucleus of microglia and neurons (Additional file 6: Figure S5B1 & B2) in the same mice presented in Additional file 5: Figure S4D & E. Indeed, TEM with no stain revealed SPION-miD2861, observed as EDNs, in the nucleus of neurons (Fig. 7F & F1), macrophages (Fig. 7G & G2), and microglia (Fig. 7H & H1). We observed several individual EDNs in Ly/Ex of microglia; there were no EDNs in the glia, either partially stained or unstained (Additional file 5: Figure S4H or Fig. 7I). The EDNs near the endothelia, identified by uranyl stain, were within the Ly/Ex of nearby cells (Fig. 7J & J1). The control (non-targeting SPION only) shows EDN scattered in the Ly/Ex (Fig. 7K). Importantly, we have no evidence showing preferential retention in any one type of cells when SPION-sODN is administered at the optimal quantities we used in MEC MRI. We investigated whether EDN retention was related to HDAC5 mRNA binding. The correlation (linear coefficiency) of HADC5 mRNA copy number and ΔR2* values by SPION-hdac5AS2 in the striatum of mice in the control (S1), acute, and chronic paradigms was 1.0 (Fig. 4c).

**Fig. 8:**
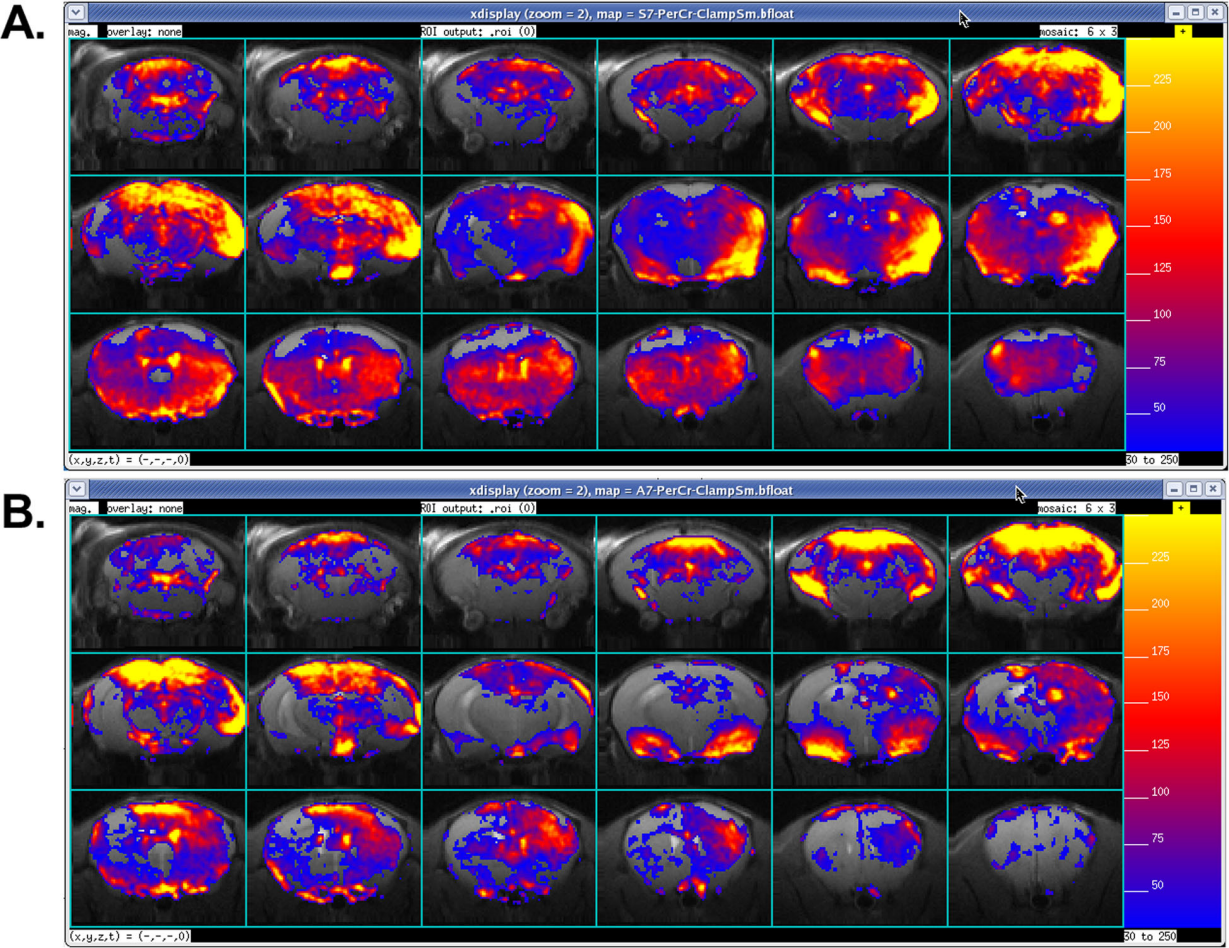
Representative ΔR2* maps (in percent increase from baseline (color scale was 0–250%) for saline control (**a**) and chronic paradigm (**b**), according to Fig. 1

**Fig. 9:**
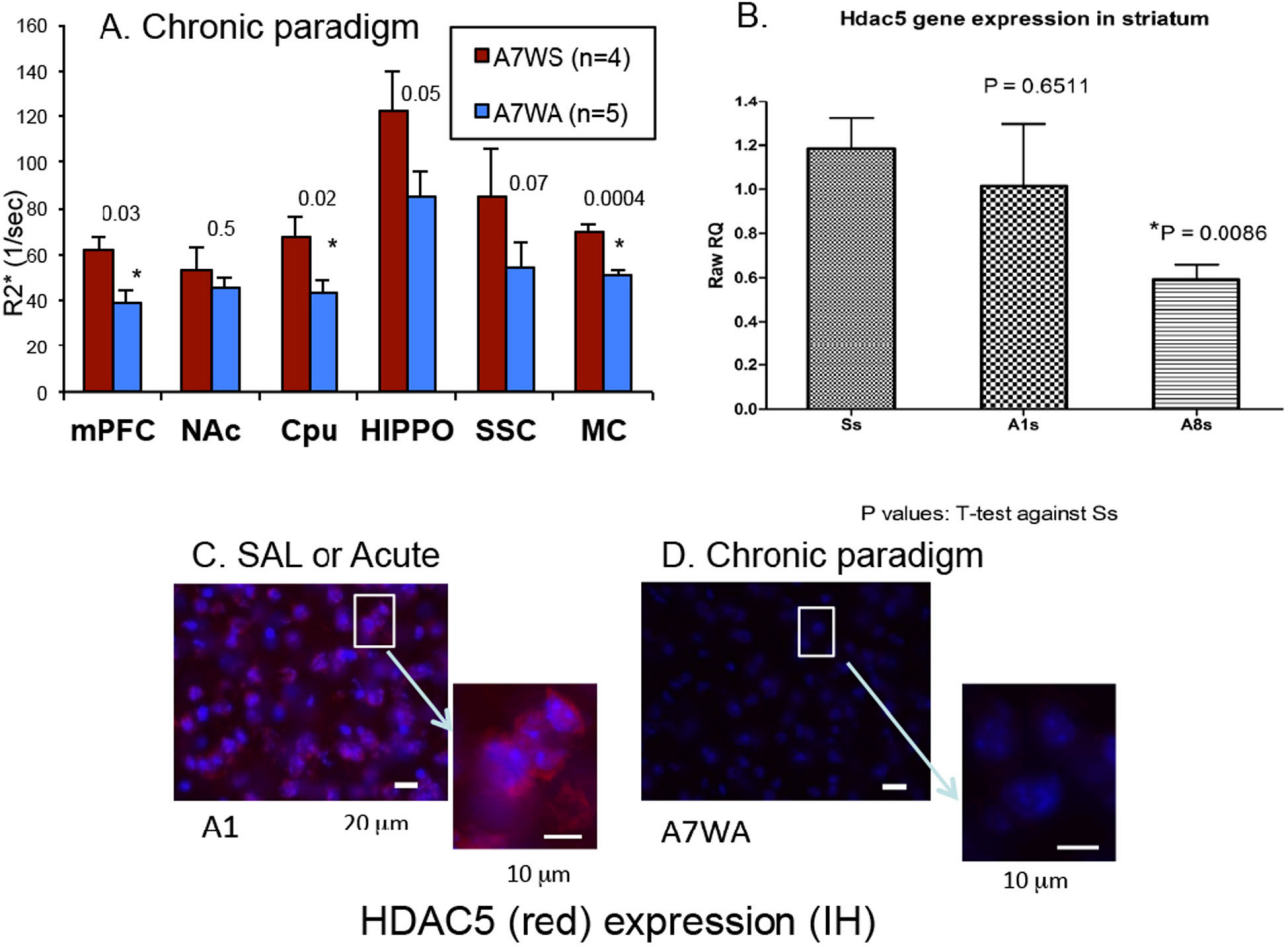
Quantitative HDAC5 expression using SPION-mid2861, MCE MRI, RT-PCR and immunohistochemistry. Panel **a** Chronic versus Control. There was a general reduction in HDAC5 mRNA measured using SPION-miD2861. The *p* values are listed above each ROI. Panel **b** The copy number of HDAC5 mRNA on the 212 region was measured from total striatal RNA of S1 (SS), A1 (A1s) and A7Wa (A8s) paradigms (*n* = 4 each) using TaqMan® analysis. Panel **c** & **d** HDAC5 protein (*red*) shows antigen around the nuclei (*purple*, DAPI) in the striatum; the intensity of total HDAC5 protein was similar in both S1 and A1 paradigms but it was null in the chronic (A7WA) paradigm

After receiving SPION-miD2861, A1 (*n* = 5) animals showed no significant differences in all ROIs of the brain compared to normal mice that received saline (the S1) (data not shown). Figure 8a, b show ΔR2* maps in the control (A7WS) and challenge (A7WA) mice of the chronic paradigm, respectively. Compared to A7WS group, mice in the challenge group (A7WA) exhibited regional reduction in HDAC5 mRNA levels. We observed significant reductions of HDAC5 mRNA in the mPFC, Cpu, and MC, but no significant attenuation in the NAc, Hippo and SSC regions (Fig. 9a). TaqMan® analysis of HDAC5 mRNA copy number in the striatum showed a decrease in the chronic exposure paradigm (Fig. 9b); there was no significant change in the control Actin mRNA in any of the three conditions (not shown). The attenuation of HDAC5 mRNA in the striatum was confirmed by a corresponding decrease in total HDAC5 antigen (Fig. 9c, b).

**Fig. 10:**
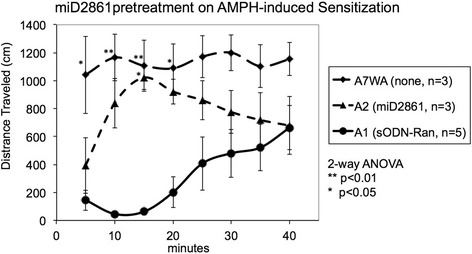
Pretreatment of miD-2861 and AIS in the acute paradigm. The distance traveled at each 5-min interval is the product of 5 cm and the summation of frequencies every 5 min

Having demonstrated that SPION-miD2861 targets and reports HDAC5 expression, we monitored changes in behavior after amphetamine. The appearance of AIS in the A1 control (sODN-Ran + A1) had a delay of onset during the first 20 min (no significant elevation) then a gradual increase during the second 20 min after amphetamine (*p* < 0.04, activities at 20 min versus 40 min). This delay was not significantly different from that seen in mice treated with saline before amphetamine [2]. In the A1 control animals AIS returned toward normal 60 min after amphetamine (not shown). On the other hand, we observed no delay to AIS in the chronic paradigm. In the first 5-min interval the A7WA group showed immediate AIS, which lasted for at least 60 min. We did not observe AIS in the A7WS and S7WS control groups (Additional file 7: Figure S6). The total distance (meters) traveled in the 40 min after amphetamine was 25 ± 9 for A1 control mice versus 90 ± 13 for A7WA mice; resulting in *p* < 0.004 per two-tailed t-test. Figure 10 shows the effect of sODN-miD2861 pretreatment on AIS during the first 20 min. Pretreatment of sODN-miD2861 in the A1 paradigm slightly but non-significantly elevated AIS at earlier time than A1 control but the attenuation in the delay was not significantly different (not shown). Pretreatment of sODN-miD2861 in the A2 paradigm attenuated the delay from 40 to 15 min. The same pretreatment to the chronic paradigm did not significantly change already sensitized locomotor behavior (not shown). Statistical analysis by two-way ANOVA shows significant difference in AIS by amphetamine and miD2861 treatments (F = 29.78, df = 3, *p* < 0.0001), with significant difference in the time of AIS delay (F = 2.585, DF = 7, *p* = 0.043).

**Fig. 11:**
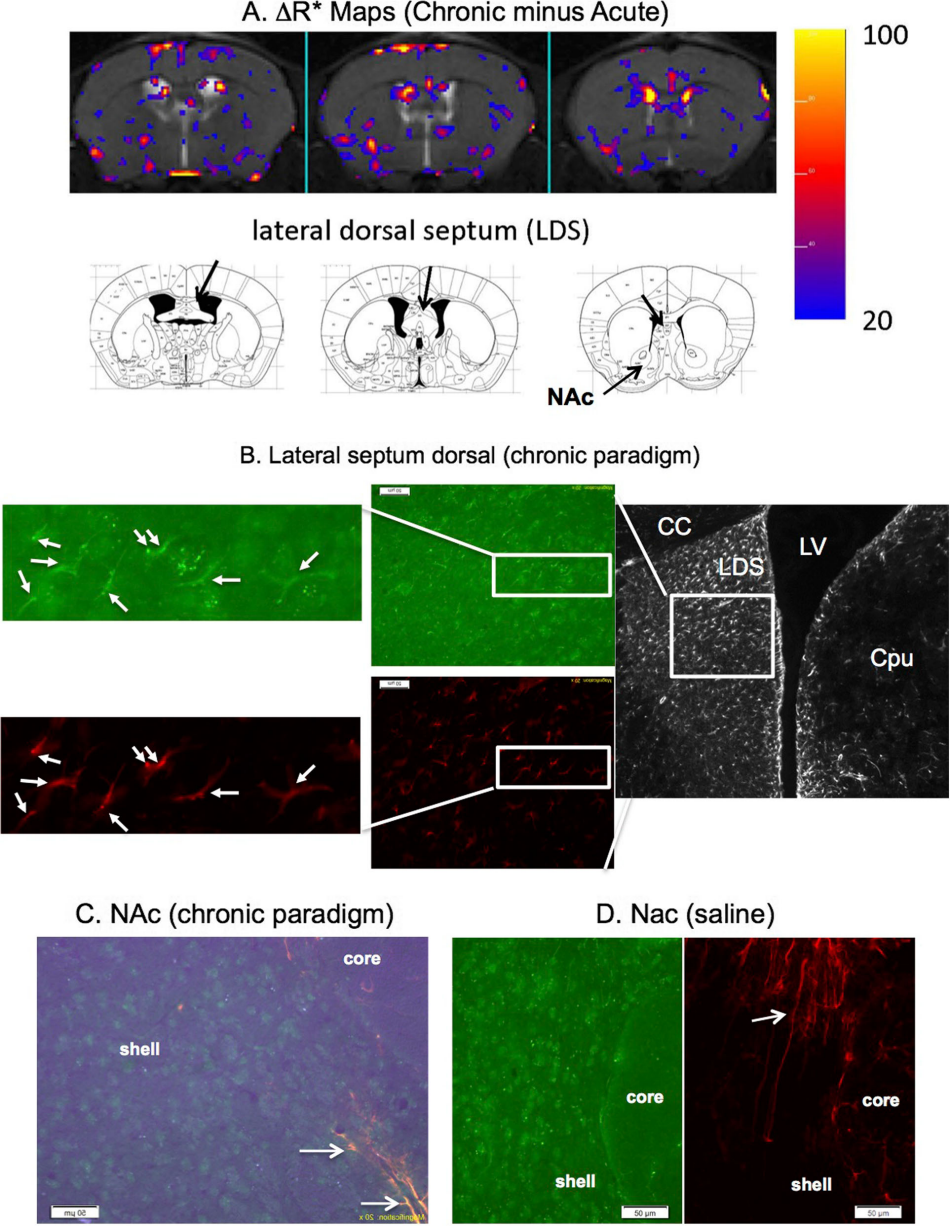
SPION-miD2861 identifies neuroprogenitor cells. Subtraction R2* maps (chronic minus acute) identify ROIs in the LS in vivo (panel **a**). We stained brain tissue using Cy3-IgG-GFAP and Cy-2-IgG-NeuN; neuroprogenitor cells were identified by co-expression of both antigens (*arrows*, Panels **b** & **c**). Panel **c** contains image spectrum (*purple*) for Cy5.5-SPION-miD2861. The *arrow* in Panel **c** shows ordinary GFAP^+^/NeuN^−^ astroglia. Corpus callosum (CC) and lateral ventricle (LV) are shown

Based on subtraction R2* maps that identified regional repair/remodeling [23, 29, 31], we are able to also identify changes in HDAC5 expression in the chronic paradigm. Figure 11a shows R2* elevation in the LS of three consecutive MR sections (a total of 1.5 mm in the dorsal LS); however, we observed several ROIs in the NAc/Striatal (arrows). We found the LS contained progenitor cells that exhibited NeuN^+^ (neuronal biomarker) and GFAP^+^ phenotype (Fig. 11b). We also found ventral striatum (Cpu) contained few progenitor cells except on the ventricular wall (Fig. 11b, black/white panel); limited progenitor cells on microvascular walls in the NAc/Striatum (arrows, Fig. 11c). Immunohistology of progenitor cells in panels B & C matched elevated ΔR2* maps in panel A. In control mouse brains, we found glial cells do not express NeuN and have long axons (Fig. 11d). We concluded that SPION-miD2861 could reliably monitor HDAC5 expression and locate neuroprogenitors based on HDAC5 expression in preclinical models of neural network remodeling.

## Discussion

We have reported here that miD2861 targets HDAC5 mRNA in living brains; our data support that a reduction in HDAC5 expression involves AIS in a preclinical model of amphetamine exposure. Moreover, SPION-miD2861 and MCE MRI identify regional repair/remodeling in the network that involves the pleasure, aggression, and reward pathways in the LS and NAc [32]. These conclusions come from studies of semi-quantitative MCE MRI in vivo, validated by ex vivo assays. We have shown that MCE MRI of the miR-2861 binding site on HDAC5 mRNA is consistent with both immunohistochemistry and RT-qPCR results. However, results from MCE MRI with a primer that does not target the miR-2861-binding site do not agree with immunohistochemistry. The differences between these results could be related to intact versus degraded HDAC5 transcripts; our HDAC5 mRNA measurements using SPION-miD2861 (MCE MRI), sODN-miD2861 (RT-qPCR) and IgG against HDAC5 agree with attenuation of intact and translatable HDAC5 transcripts. Therefore, the evidence we show here provides solid support to the hypotheses that SPION-miD2861 reports HDAC5 expression, which leads to mechanistic investigation of AIS and neuroprogenitor monitoring.

Acetylation patterns in chromatin affect gene transcription and induce drug sensitization following chronic exposure to drugs of abuse. Among the mechanisms that have been proposed to mediate exposure to drugs of abuse are increases in immediate early genes and HDAC5 shuttling [33, 34], as well as a reduction in monoamine oxidase A by proteins of immediate early genes [2]. HDAC inhibitors are used therapeutically in the psychiatry and neurology fields as mood stabilizers, antidepressants, and anti-epileptics [35, 36], for neuroprotection from ischemic injury and anti-inflammatory responses [37–42], as well as for neural remodeling [6, 11, 43]. Transport of phosphorylated HDAC3 protein from nucleus to cytoplasm is a known mechanism of drug sensitization [13, 44, 45]. However, we have presented evidence here that attenuation of HDAC5 (mRNA and antigen) mediates early appearance of AIS in the acute paradigm with miD2861 pretreatment and chronic paradigm. The results thus support the proposal that miR-2861 may reduce translatable HDAC5 mRNA [15]. In this study, we identified neuroprogenitors in the LS. Neuroprogenitor cells may be associated with neural plasticity [43], and therefore, may be active in neural network remodeling in brain repair after stroke [46]. Amphetamine has been shown to induce neuronal division (neurogenesis) in the hippocampus [47]. We have not measured neurogenesis using S-phase markers; however, we have previously monitored neuroprogenitors using SPION-nestin and SPION-gfap. Here we expanded the functional applications of MCE MRI by precise delivery of SPION-miD2861. Target specificity of small molecules with signal sensitivity will further enable monitoring of cerebral repair and remodeling in vivo.

We have applied SPION to label miD2861. Iron oxide NPs are commonly used for stem cell labeling [48]; the SPION we have used is at much lower dose than that used in stem cell labeling. Our earlier studies used SPION with a hydrodynamic diameter (dia) of 20 ± 10 nm and molecular weight (as SPION-cfos for Fos mRNA) of 5.6 × 10^−19^ g Fe per NP. Each SPION-cfos contains ~6000 iron atoms, based on the relative viscosity of SPION in sodium citrate buffer at pH8 [20]. For our current studies we use commercially available dextran-coated iron oxide, which provides strong T2 contrast, with a similar number of Fe molecules per particle (1.5 × 10^−20^ mol Fe per NP). Molday Ion has an average hydrodynamic size of 30 ± 20 nm (Zetasizer, Malvern Instruments) and a core size of 8 ± 5 nm. Long-term use of these NPs does not change liver enzyme levels or induce oxidative stress [49, 50]. Moreover, we have observed transient passage of iron through the hepatic system; there is no hepatic retention of iron until 10 h after i.p. delivery to mice with an icv port. [23] Whereas our previous studies used the icv route alone to deliver NPs, which was associated with an accumulation of excess macrophages at the icv puncture site [51], we have modified our methods in rodents to use a small icv port for BBB bypass. This new delivery route is noninvasive, and yields more uniform R2* values as well as eliminating macrophage accumulation.

We have observed retention of NPs of approximately 30 nm (dia) in the nuclei of neurons, microglia, macrophages, and/or phagocytes when uptake plateaus between 4 and 6 h after SPION delivery. Once the NP reaches neural cells, the cytoplasmic EDNs in our studies could be transported via a membrane-bound mechanism associated with the ER (Additional file 6: Figure S5A1), and then to the nucleus, perhaps through the nuclear pore complex associated with the ER [52, 53]. Because we identified SPION-miD2861 in TEM without staining, we have not identified endosomes before reaching the ER. The presence of scattered EDNs in Ly/Ex after SPION-miD2861 delivery suggests these EDNs are being degraded or excluded in Ly/Ex to microvessels (Fig. 7J1 & K). Most important, we have no evidence of preferential retention in any type of neural cells when SPION-sODN is administered at the optimal quantities we use in MEC MRI.

Cell typing using NeuN and GFAP for progenitor cells that are identified using miD2861 show the NeuN+ and GFAP+ phenotypes in progenitor cells (Fig. 11). Our results show that HDAC5 mRNA may be present in cells that express NeuN+ (neurons), GFAP+ (astroglia), or IBA1+ (activated microglia). The TEM data in Fig. 7F and Additional file 5: Figure S4G, showing EDNs in neurons, do not contradict this conclusion. Our observation differs from others’ reports that non-targeting iron oxide NPs are preferentially retained in phagocytes or macrophages of hepatic RES within one hr of delivery [54, 55]. Non-targeting iron NPs are retained in the Lys and eventually degraded [56].

Our HDAC5 mRNA-targeting NPs reports HDAC5 expression by binding to its target, as demonstrated by the linear coefficiency between MR-derived ΔR2* values and mRNA copy number quantified by TaqMan analysis (Fig. 4). There are several important factors that inform and provide rationale for using correlation studies of ΔR2* values and TaqMan analysis. First, the peak (or perhaps more precisely, plateau) ∆R2* values are positively proportional to regional iron content [22]. Second, the CNR in all ROIs of the brain is high and uniform at the plateau [29]. Third, in preparing SPION-sODN we conjugated the targeting sODN and SPION reporting agent at a constant ratio of 3 nmols sODN per mg iron. Fourth, the linkage between SPION and sODN is strong and biologically stable (via NeutrAvidin and biotin), and the use of a common transfection reagent (Lipofectamine 2000) facilitates and protects SPION-sODN in body fluids during delivery. Some SPION-sODN may enter the nuclei where the target RNA transcripts are present for binding (Fig. 7). Fifth, we use ΔR2* values at plateau retention, or 6 h after SPION-sODN delivery; the plateau ∆R2* represents strong CNR, and a steady state of RNA-bound SPION-sODN at physiological conditions during MRI. Sixth, the mRNA copy number measured under normal conditions represents the equilibrium between synthesis and degradation in the physiological condition at the time of RNA isolation. We have shown SPION and antisense sODN are intact in vivo, as SPION-sODN can be used to produce cDNA for amplification by in situ RT-PCR [51]. The correlation coefficiency between the plateau ΔR2* of cerebral FosB, HDAC5, and GFAP mRNA and copy numbers by TaqMan® Gene Expression Assay (with Actin mRNA as reference) supports precision hybridization as the mechanism of MCE MRI reporting mRNA, as validated by RT-PCR. Last but not the least, ΔR2* values of SPION-sODN representing a steady state of RNA-bound iron oxide in the nucleus is supported by the static dephasing regime theory of iron oxide compartmentalization [57–59].

Some studies have reported advanced fluorescent in situ hybridization (FISH) of miRNA [60]. Short interfering nucleic acids (siDNA or siRNA) can be used in targeted molecular imaging for theranostic applications [18, 61]. The core size of SPION-sODN of 8 *±* 5 nm (dia) is within the pore size of the nuclear envelope for passage [62]. Although the mechanism of precision translocation of SPION-sODN to nuclear HDAC5 mRNA in the neural environment is not yet well understood, neural cells may have intrinsic repair mechanisms to meet the required transport and exclusion of unbound or excess SPION-sODN, and preferential protection of bound SPION-sODN under normal physiologic temperature. These mechanisms must work in concert in vivo for various physiological functions. The positive correlation between MR-derived ΔR2* values and mRNA quantitation by RT-PCR suggests that ΔR2* values from MCE MRI report iron, predominately, hybridized to target mRNA; this in turn supports the view that one optimal dose of target-specific NPs does not yield more EDNs in Ly/Ex than seen in the control (Additional file 2: Figure S1). Therefore, the exclusion of unbound targeting NPs is rapid, within 4 h after delivery to living mouse brains. However, we must note that we cannot estimate copy number from MRI because the current technology does not provide an internal control for copy number estimation.

We have also examined for possible change in miR2861 level. When comparing across the three paradigms we detected no significant differences in the level of miR2861 (not shown). This is in agreement with reports that no detectable miR2861 is found in adult brains [15]. The main function of the class of small single-stranded noncoding RNAs known as miRNA is to regulate mRNA translation; this occurs in normal developmental as well as in pathological conditions across the general population, in all ages, genders, and races. By regulating mRNA translation, miRNA affects differentiation of normal biological development, resistance to environmental changes, cellular proliferation and apoptosis; it may also have dire consequences, causing an array of health problems. Although they were once mistakenly termed “junk” RNA, we now know miRNAs play an important role in epigenetic pathways. The human genome has thousands of miRNAs that target about 600 genes. The availability of miRNA can also help stabilize and protect cytoplasmic enzymes. All cells express miRNA, starting with precursor miRNAs, which are transcribed as part of one arm of a ~80 nucleotide RNA stem-loop by RNA polymerase II or III. These precursors are post-transcriptionally processed to pre-miRNA by Digeorge Syndrome Critical Region 8 (DGCR8), a nuclear splicesome with a de-branching enzyme, and the catalytic ribonuclease (RNase) III domain, which liberates a hairpin loop structure of about 70 nucleotides each of precursor miRNA. Mirtrons directly splice out introns of some pre-miRNAs, which are composed of a matured sequence and a passenger sequence on the hairpin and a two-nucleotide overhang at the 3′-hydroxyl end. Exportin-5, a nucleocytoplasmic shuttle, exports pre-miRNA from the nucleus by an energy-dependent process. The Dicer enzyme with RNase III activity in the cytoplasm produces an imperfect RNA: RNA duplex of about 22 nucleotides in length, by cutting away the loop in the hairpin of pre-miRNA. The RNA-induced silencing complex (RISC) incorporates one miRNA strand in the duplex based on thermodynamic instability and weaker base pairing relative to the other strand. The passenger strand can be incorporated into another RISC for a different RNA target, but is generally preferentially degraded. The Argonaute (Ago) family of proteins provide the active part of miRISC’s (RISC with miRNA) silencing function to block translation. Because extensive ex vivo purification is used to detect miRNA, how RISC may protect miRNA in vivo for detection is not well understood. What is known is that the Ago family encodes four functional domains: the N-terminal, PAZ, Mid, and C-terminal PIWI domains. The PAZ domain (a conserved domain of PIWI, Ago and Zwille proteins) binds to the single-stranded 3′ end, while the RNase-H activities of the PIWI (P-element induced wimpy testis) domain cleave the target mRNA. Gene silencing occurs within miRISC, between the matured strand of miRNA and its target mRNA. Perfect complementarity in the seed region, at nucleotides 2–7 of miRNA, will speed up degradation of the target mRNA and allow transient gene silencing, manifesting as a gene knockdown phenotype; therefore, a perfect match up between matured miRNA and target mRNA is not required to inhibit translation. The addition of a methyl moiety (in plants) or an adenine residue to the 3′ end of the miRNA protects or stabilizes the matured miRNA.

Amphetamine-type stimulants (e.g., methamphetamine or 3-4-methylenedioxy methamphetamine (MDMA), also known as Ecstasy or MOLLY) are highly addictive Schedule I drugs, and widely abused worldwide (2011 Global ATS Assessment, a United Nations report). Those who habitually use MDMA are at high risk for learning impairment and aggressive behavior [63], as well as other symptoms that mimic those seen in schizophrenia and depression [64, 65]. Further compounding the health issues associated with drug addiction is that, given the propensity for needle sharing, drug addicts face a highly increased risk of infection from blood-borne pathogens such as human immunodeficiency virus type 1 (HIV-1). Significant variations in brain structure are associated with both HIV infection and methamphetamine dependence [66]. This relationship is all the more significant because deacetylation of histone within the HIV-1 long terminal repeat by HDACs helps to maintain viral latency, allowing the virus to evade both immune detection and antiviral drugs [67]. Methamphetamine has been found to induce regional variations in oxidative stress and behavioral modifications in HIV-1Tg rats [68–70]. Moreover, non-selective inhibitors of HDAC may induce HIV-1 expression from the HIV-1 reservoir in resting CD4(+) T cells, without new infection [71]. As such reservoirs are also known to be present in the microglia of the CNS, the technology we have developed to monitor transcripts in microglia promises additional significance as a tool to monitor viral latency and drug addiction longitudinally. Understanding mechanisms of HDAC expression and neural remodeling in vivo are of great importance, with broad implication for public health.

## Conclusions

We designed sODN-miD2861 to target HDAC5 mRNA; our binding assays and primer are specific to the location of miD2861 binding ex vivo and in vivo. Our MCE MRI reports RNA-bound NPs. The linear regression of coefficient of MRI and TaqMan® analysis for copy number was near 1.0 at different conditions, demonstrating that the mechanism of gene targeting for SPION-sODN and MRI is similar to that of RT-PCR. Because HDACs or cells that harbor them are involved in cerebral repair/remodeling processes, changes in HDAC5 expression, and the availability of tools to monitor such change, may have still broader implications than we have explored in the scope of this work.

## Additional files

Additional file 1: Supplemental Material. (DOCX 85 kb)
Additional file 2: Expression of HDAC5 antigens in the nucleus accumbens (NAc) of naïve mice mice. We compared total (cy3-ab1439, Abcam) or phosphorylated HDAC5 (cy3-ab192339) in the nucleus accumbens (NAc). (PDF 1 kb)
Additional file 3: Expression of HDAC5 antigens in mice of acute amphetamine exposure groups. We compared total (file 2) or phosphorylated (file 3) HDAC5 in the NAc. (PDF 3461 kb)
Additional file 4: Expression of HDAC5 antigens in mice in the chronic amphetamine exposure groups. We compared total (file 4) or phosphorylated (file 5) HDAC5 in the NAc. (PDF 2600 kb)
Additional file 5: We observed electron dense nanoparticles (EDN) in mice with (D, E, G & H) or without (A-C) SPION-sODN at the optimal dose (0.04 mg/kg, intracerebroventricular [icv] injection); there is no preferential accumulation of EDNs in mice with optimal dose of SPION-sODN. Accumulation of EDNs are observed in mice with 3X optimal dose (0.12 mg/kg, intracerebroventricular injection, not shown) or 8 optimal doses at one injection every week (4 mg/kg, intraperitoneal [i.p] injection, F). We attempted to identify SPION-sODN of 30 nm (dia) in Fig S4E; TEM stains masked our ability to identify SPION-sODN as EDN at ~30 nm (dia). (PDF 5888 kb)
Additional file 6: We observed several nuclear EDNs with a uniform diameter of 30 nm (A1, B1 & B2, arrowheads); three of these EDNs appeared on the membrane in tandem near the rough ER (A1). Only EDN larger than 60 nm appeared to be in the cytoplasm (B & B1, arrows). These unstained samples had reduced background noise, and we found EDNs (arrows) in the cytoplasm and nuclei. Although EDNs were visible, we cannot identify Ly/Ex, but can identify MG and N from the outline of their nuclei. Bars (microns) = 500 (A, A1 & B). (PDF 2836 kb)
Additional file 7: We conducted a gross comparison of the total locomotor activities, and compared changes in locomotion between AMPH-treated and saline-treated animals in various exposure paradigms (SAL vs. AMPH groups in acute exposure, SAL7/W/S vs. A7/W/A and SAL7/W/S vs A7/W/S [placebo] in chronic exposure groups). Using one-way ANOVA followed by Newman-Keuls Multiple Comparison test, we found that AMPH induced a significant main effect (p < 0.001), with an exception between the SAL7/W/S and A7/W/S groups (p > 0.05). The average rate of locomotion (in meters per hour) was 57 + 6 and 105 + 6 for A1 and A7/W/A, respectively. (PDF 107 kb)

## Abbreviations

A: Amphetamine
AIS: Amphetamine-induced sensitization
B_0_: External magnetic field
bp: base pairs
CC: Corpus callosum
Cpu: Caudate putamen
EDN: Electron-dense nanoparticles
Ex: Exosome
GFAP: Glial fibrillary acidic protein
GFP: Green fluorescent protein
HDACs: Histone deacetylases
hipp: hippocampus
i.p.: intraperitoneal
IBA1: Ionized calcium-binding adaptor molecule 1
icv: intracerebroventricular
LS: Lateral septum
LV: Lateral ventricles
Ly: Lysosome
M: Monocytes
MC: Motor cortex
MG: Microglia
mPFC: medial prefrontal cortex
MRI: Magnetic resonance imaging
N: neuron
NA: NeutrAvidin
NAc: Nucleus accumbens
NP: Nanoparticles
P: Phagocytes
R2*: The effective rate of transverse relaxivity
ROI: Region(s) of interest
RT-PCR: Reverse transcription polymerase chain reaction
SPION: Superparamagnetic iron oxide nanoparticle
SSC: Somatosensory cortex
ΔR2*: Above the background transverse relaxivity
