# Supplementary material for: Epigenetics of amphetamine-induced sensitization: HDAC5 expression and microRNA in neural remodeling
Source: J Biomed Sci. 2016 Dec 8;23:90. doi: 10.1186/s12929-016-0294-8 (PMC5146867; doi:10.1186/s12929-016-0294-8)
Supplement: Additional file 1: — Supplemental Material. (DOCX 85 kb) [file 12929_2016_294_MOESM1_ESM.docx]

Supplemental Material

Title: **Epigenetics of Drug Sensitization: MicroRNA and HDAC5 expression in neural remodeling by amphetamine**

Short title: **Target-guided Delivery and MRI Quantitation in vivo**

Philip K Liu* and Christina H. Liu^1^

Molecular Contrast-Enhanced MRI Laboratory at the Athinoula A. Martinos Center for Biomedical Imaging, Department of Radiology, Massachusetts General Hospital and the Harvard Medical School

Charlestown, Massachusetts 02129

^*^Corresponding Author:

Philip Liu, PhD

Associate Professor of Radiology

CNY149 (2301) Thirteenth Street

Charlestown, MA 02129

Office: 617-724-4563

Fax: 617-726-7422

[philipl@nmr.mgh.harvard.edu](mailto:philipl@nmr.mgh.harvard.edu)

^1^ co-author e-mail:

Christina H. Liu, PhD. PE.

<chliu1104@yahoo.com>

Methods

**Amphetamine exposure paradigms**: For the chronic exposure paradigm, eight age-matched, amphetamine-naïve, male C57black6 mice received a single dose of amphetamine in their home cage every other day, for a total of seven injections of amphetamine (4 mg/kg, by injection intraperitoneally [i.p.] A7) ([1-5](#_ENREF_1)); this was followed by two weeks with no drug exposure (abstinence, A7W in Fig 1A). A final dose of amphetamine or saline was given on the day of post-SPION MRI, such that we could compare the effect of a challenge dose of amphetamine following chronic exposure and abstinence in our A7WA group to control groups without challenge dose (A7WS or S7WS). For the acute exposure paradigm (A1), age-matched, amphetamine-naïve, male C57black6 mice received a single dose of amphetamine (4 mg/kg, i.p.); the control group received a single dose of saline (S1, vehicle, 10 ml/kg, i.p.).

**Immunohistochemistry of total and phosphorylated HDAC5 antigens in naïve, acute and chronic amphetamine exposure paradigms:** For ex vivo assays we treated 2 C57black normal mice with A7W paradigms; we treated 4 normal naïve mice with saline or amphetamine with acute paradigm (n = 4 each) but all these 6 mice received no icv puncture nor contrast agent. We administered saline (100 μl, i.p) or amphetamine (4 mg per kg, i.p) three hours before the mice (n > 2, each group) were put under general anesthesia and retrograde-perfused with ice-cold saline. Isolating brain tissue as described ([6](#_ENREF_6)), we stained slices of brain tissue (25 micron in thickness) with total or phosphorylated HDAC5 antigens with ab1439 (Abcam) and ab192339 (phosphor S529), respectively. We then co-stained Cy2-labeled rabbit polyclonal IgG against glial fibrillary acidic protein (GFAP, Z0334, Dako) or Cy3-labeled polyclonal IgG against ionized calcium-binding adaptor molecule 1 (IBA1, ab5076, Abcam); nucleic acids were stained with DAPI (1:500 dilution) ([1](#_ENREF_1)). To examine HDAC5 mRNA expression using Cy3-sODN (see below), transgenic mice (n=3) underwent icv puncture one week before they were administered Cy3-sODN (120 pmol in 0.1 ml, ip) and a dose of amphetamine, as in the acute paradigm (Fig 1B). All histological images were acquired using the same exposure time and gain, using a Retiga EXi camera on an Olympus microscope and cellSens Dimension software (Olympus America Corp, Nashua, NH).

**Biotinylated sODN for HDAC5 transcripts:** We designed two sODNs with antisense sequence to HDAC5 mRNA; for all sODNs we used nucleotide BLAST to validate target mRNA with potential binding (<http://blast.ncbi.nlm.nih.gov/Blast.cgi>). The sequences were obtained from GenBank (AF207748): sODN of HDAC5 mRNA or miR-2861 binding site (hdac5).

**Modular contrast agent using SPION-NA:** We synthesized NeutrAvidin (NA)-labeled Molday Ion (CL-30Q02-2, BIOPAL, Worcester MA) using the protocol previously published ([7](#_ENREF_7)). This commercially available Molday Ion contains 6k-9k molecules of iron oxide (dextran-coated superparamagnetic iron oxide nanoparticles or SPION), and has **a unique Zeta potential (-5 mV)** with an effective size of 25 nm (dia) and relaxivities of R1 = 15.4 and R2 = 33.9 s^-1^mM-^1^.

**Validation of SPION-sODN delivery using transmission electron microscopy (TEM)**: We collected tissue samples immediately after MRI; the left NAc of S1 and A1 mice was immersed in 2.5% PBS-buffered glutaldehyde at 4^º^C and sent to the TEM laboratory of the Histology Core Facility at the MGH Center for Systems Biology for preparation and double-blinded examination. After tissue was dehydrated in ascending concentrations of ethanol, immersed in propylene oxide, and embedded in Epon 812 resin (Agar Scientific Ltd., Standstead, England), samples were cut into ultrathin sections (~60 nm). The Core prepared tissue with and without standard TEM stain using osmium tetroxide (1%, 2 hrs), uranyl acetate (Ua, 2%, 5 min) and Reynold’s lead citrate ([20](%2523_ENREF_20)). We found that standard staining masked NP identification; we modified TEM staining by omitting all stains, unless indicated, to reduce the background of membrane structure and enable visualization of SPION. The neuronal nucleus was identified as a smooth, round nuclear body with diameter of ~7 microns. We defined microglia (MG) by the presence of irregular euchromatic nucleus, a peripheral rim of heterochromatin, and various empty and partially filled lysosomes/exosomes (Ly/Ex).

**Locomotor Assessment.** We measured locomotor behavior modification according to drug sensitization protocols ([1-5](#_ENREF_1)). To measure horizontal locomotion and fine motor activity, we used an automated recording device (San Diego Instrument, San Diego, CA) located in the same room in which the animals were individually housed. The system has eight chambers, each of which is composed of frames equipped with five infrared photocell beams (spaced 5cm apart) in one polypropylene cage (15 x 25 cm). The photocell beams traverse each cage in a plane above the floor. We recorded the frequency of locomotion (ambulation) as the number of sequential breaks in two adjacent beams and measured fine motor activities (such as grooming or other stereotyped motions) by counting the number of sequential breaks in a single beam. Recordings were made every minute for at least 60 minutes; we reported the distance traveled in every 5 min as the product of 5 cm and the summation of frequencies of beam break in the respective 5 min interval.

Mice were located in the same room in which eight mice were individually housed and tested in their own home cages. We pre-conditioned the mice by removing them from and returning them to their cage daily for five days prior to behavior assessment. To examine the effect of HDAC5 knockdown, we pretreated mice with a dose of miD2861 or placebo (sODN with random sequence, or sODN-Ran) at 1.2 mmol/kg (i.p./icv) three hours before administering amphetamine to naïve (A1) mice or mice that had been previously exposed to one dose of amphetamine (A2) or A7W), as previously described ([1](#_ENREF_1), [8](#_ENREF_8)). We performed locomotor assessment immediately, as described above. We obtained data from twice the number of mice calculated by power analysis. We computed the mean and SEM from the average values in each group of animals, and compared the statistical significance of these values using a *t* test (two tail, type II or equal variant) or two-way ANOVA (GraphPad Prism IV, GraphPad Software, Inc., San Diego, CA). A *p* value of < 0.05 was statistically significant

**References**

1. Liu, C. H., Ren, J. Q., Yang, J., Liu, C. M., Mandeville, J. B., Rosen, B. R., Bhide, P. G., Yanagawa, Y., and Liu, P. K. (2009) DNA-based MRI probes for Specific Detection of Chronic Exposure to Amphetamine in Living Brains. *J Neurosci* **29**, 10663-10670

2. Giordano, T. P., 3rd, Satpute, S. S., Striessnig, J., Kosofsky, B. E., and Rajadhyaksha, A. M. (2006) Up-regulation of dopamine D(2)L mRNA levels in the ventral tegmental area and dorsal striatum of amphetamine-sensitized C57BL/6 mice: role of Ca(v)1.3 L-type Ca(2+) channels. *J Neurochem* **99**, 1197-1206

3. Xue, C. J., Ng, J. P., Li, Y., and Wolf, M. E. (1996) Acute and repeated systemic amphetamine administration: effects on extracellular glutamate, aspartate, and serine levels in rat ventral tegmental area and nucleus accumbens. *J Neurochem* **67**, 352-363

4. Paulson, P. E., and Robinson, T. E. (1991) Sensitization to systemic amphetamine produces an enhanced locomotor response to a subsequent intra-accumbens amphetamine challenge in rats. *Psychopharmacology (Berl)* **104**, 140-141

5. Paulson, P. E., Camp, D. M., and Robinson, T. E. (1991) Time course of transient behavioral depression and persistent behavioral sensitization in relation to regional brain monoamine concentrations during amphetamine withdrawal in rats. *Psychopharmacology (Berl)* **103**, 480-492

6. Liu, C. H., Ren, J., Liu, C. M., and Liu, P. K. (2014) Intracellular gene transcription factor protein-guided MRI by DNA aptamers in vivo. *FASEB journal : official publication of the Federation of American Societies for Experimental Biology* **28**, 464-473

7. Liu, C. H., Kim, Y. R., Ren, J. Q., Eichler, F., Rosen, B. R., and Liu, P. K. (2007) Imaging cerebral gene transcripts in live animals. In *J Neurosci* Vol. 27 pp. 713-722

8. Liu, C. H., Ren, J., and Liu, P. K. (2016) Amphetamine manipulates monoamine oxidase-A level and behavior using theranostic aptamers of transcription factors AP-1/NF-kB. *J Biomed Sci* **23**, 21

**Figure legends**

Figure S1-S3. Expression of HDAC5 antigens in mice in the naïve (saline), acute and chronic amphetamine exposure groups. We compared (A) total (cy3-ab1439, Abcam) or (B) phosphorylated HDAC5 (cy3-ab192339) in the nucleus accumbens (NAc) of mice in the saline (saline, S1A & S1B), acute amphetamine exposure (S2A & S2B) or chronic amphetamine exposure (S3A & S3B) paradigms. Brain tissues were obtained one hour after amphetamine or saline (ip) in isopropanol on dry ice. Brain slides were stained with Cy3-IgG then Cy2-gfap (Z0334, Daka), and DAPI for nucleus.

Figure S4. We obtained two adjacent brain slices (0.5 mm in thickness) from the nucleus accumbens (NAc, see Mouse Brain [The Mouse Brain in Stereotaxic Coordinates, Proxinos-Franklin, Academic Press, 2001]) of mice with and without SPION-sODN (n=2 each); samples were quickly submerged in 2.5% buffered glutaraldehyde (4^o^C) after excision. Specimens were delivered to the TEM laboratory of the Microscopy Core at the MGH. After they were dehydrated in ascending concentrations of ethanol, immersed in propylene oxide, and embedded in Epon 812 resin (Agar Scientific Ltd., Standstead, England) samples were cut into ultrathin sections (~60 nm). One of each set of two brain slices was fully stained (4% uranyl acetate, osmium tetroxide and Reynold’s lead citrate), and examined with a Philips Morgagni 268 transmission electron microscope, Panel A - C). The neuron (N) was identified with a nucleus as a smooth, round nuclear body with a diameter of ~7 microns (A). We defined microglia (MG) by the presence of irregular euchromatic nucleus, a peripheral rim of heterochromatin (B), and various empty and partially filled Ly/Ex. Phagocytes (P), monocytes or macrophages (M) were identified from the typical irregular nucleus.

We readily identified lysosomes (A1 & A2) or exosomes (B1 & C1) in neurons, microglia and macrophages of mice without SPION (A - C). We observed dark electron dense nanoparticles (EDNs), perhaps from endogenous metabolites including iron, present in Ly/Ex of normal brain. One lysosome appeared to be near the rough endoplasmic reticulum (rER), but the ER was not connected to the lysosome (A1, arrow). The size of Ly/Ex ranged from 100 – 500 nm (dia).

We created a BBB bypass port by icv puncture, and delivered SPION-sODN (4 mg/kg, ip) to mice (n=2). The mice received amphetamine (4 mg/kg, ip) two hrs later, and one hr after amphetamine we obtained brain samples. We stained samples without osmium (D & F) or lead phosphate (E). SPION-sODNs were taken up via pinocytosis (D, arrow) via MG near the icv port. We observed EDNs within lysosomes (Ly, E1) and exosomes (Ex, E2) in neurons (E) from mice that received one dose of SPION-sODN, and the exosome remained connected to the rER (arrow). However, we identified multiple lysosomes in the cytoplasm of phagocytes (F, arrows) in mice that had been given repeated dose SPION-sODN (8 doses of 4 mg/kg, ip). In normal mice (n = 2) that received SPION-miD2861 (4 mg/kg, ip/icv) four hr before sample collection, we observe EDN in the Ly/Ex (G1, arrows) in neurons (G) or microglia (H). Bars (microns) = 2 (A, C, E - H); 0.5 (B, C1, D, E1 & H1); 0.1 (A1, E2).

Figure S5. We attempted to identify SPION-sODN of 30 nm (dia) in unstained samples from two mice that presented in Fig S4E); these unstained samples had reduced background noise, and we found EDNs (arrows) in the cytoplasm and nuclei. We observed several nuclear EDNs with a uniform diameter of 30 nm (A1, B1 & B2, arrowheads); three of these EDNs appeared on the membrane in tandem near the rough ER (A1). Only EDN larger than 60 nm appeared to be in the cytoplasm (B & B1, arrows). Although EDNs were visible, we cannot identify Ly/Ex, but can identify MG and N from the outline of their nuclei. Bars (microns) = 500 (A, A1 & B).

Figure S6. We conducted a gross comparison of the total locomotor activities, and compared changes in locomotion between AMPH-treated and saline-treated animals in various exposure paradigms (SAL vs. AMPH groups in acute exposure, SAL7/W/S vs. A7/W/A and SAL7/W/S vs A7/W/S [placebo] in chronic exposure groups). Using one-way ANOVA followed by Newman-Keuls Multiple Comparison test, we found that AMPH induced a significant main effect (p < 0.001), with an exception between the SAL7/W/S and A7/W/S groups (p > 0.05). The average rate of locomotion (in meters per hour) was 57 + 6 and 105 + 6 for A1 and A7/W/A, respectively.
