# Supplementary figures and images for: Epigenetics of amphetamine-induced sensitization: HDAC5 expression and microRNA in neural remodeling
Source: J Biomed Sci. 2016 Dec 8;23:90. doi: 10.1186/s12929-016-0294-8 (PMC5146867; doi:10.1186/s12929-016-0294-8)

Naïve (Saline, NAc No. 99)

Anti-HDAC5 antibody (ab1439)

Anti-HDAC5 (phospho S259) antibody ab192339

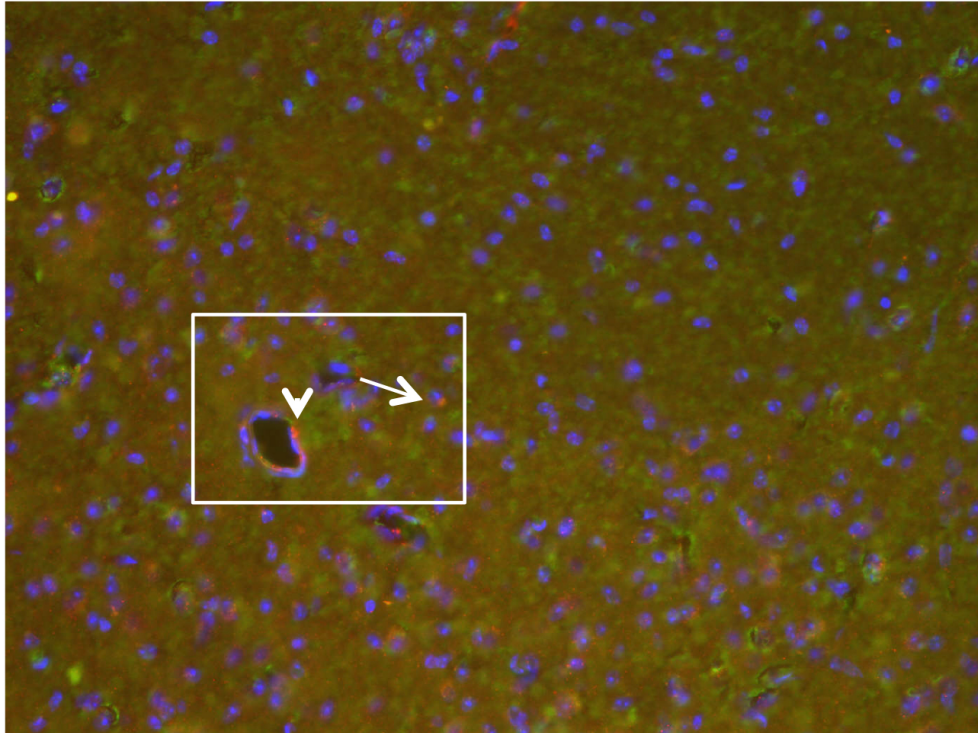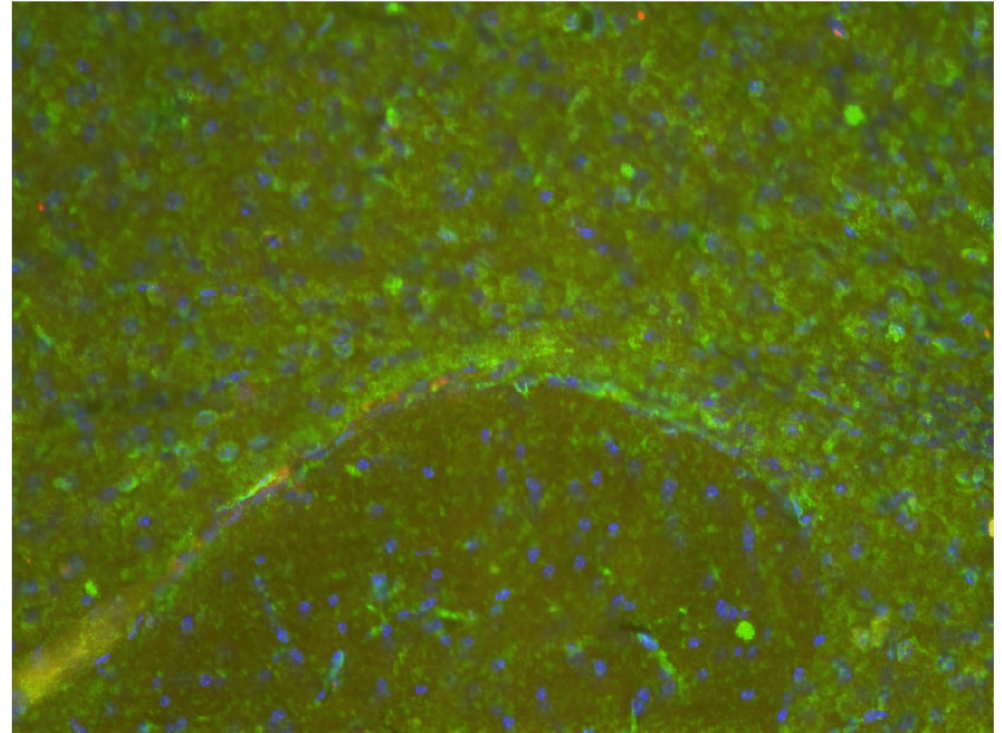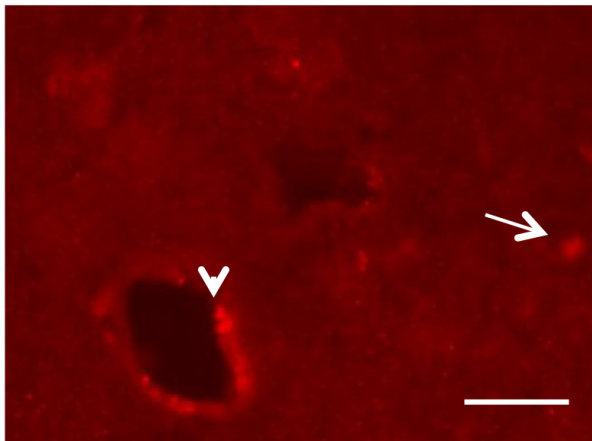

bars = 10  $\mu$ m

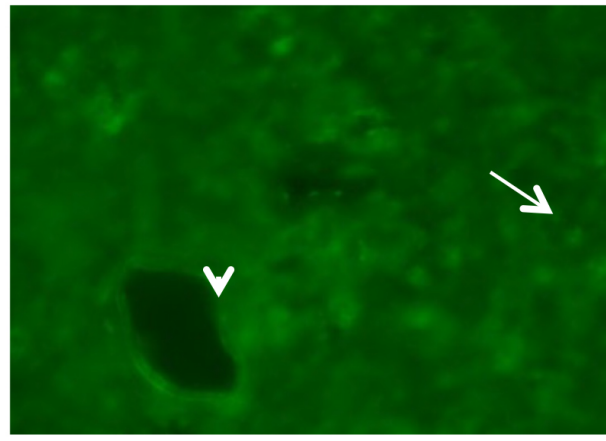

GFAP IgG (Z0334, DAKA)

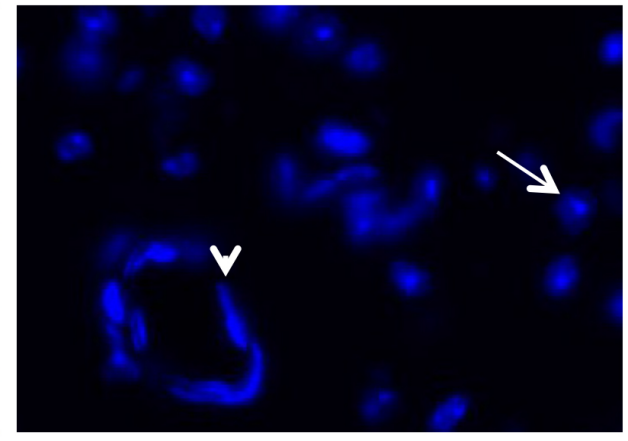

Supplement: Additional file 2: — Expression of HDAC5 antigens in the nucleus accumbens (NAc) of naïve mice mice. We compared total (cy3-ab1439, Abcam) or phosphorylated HDAC5 (cy3-ab192339) in the nucleus accumbens (NAc). (PDF 1 kb) [file 12929_2016_294_MOESM2_ESM.pdf]

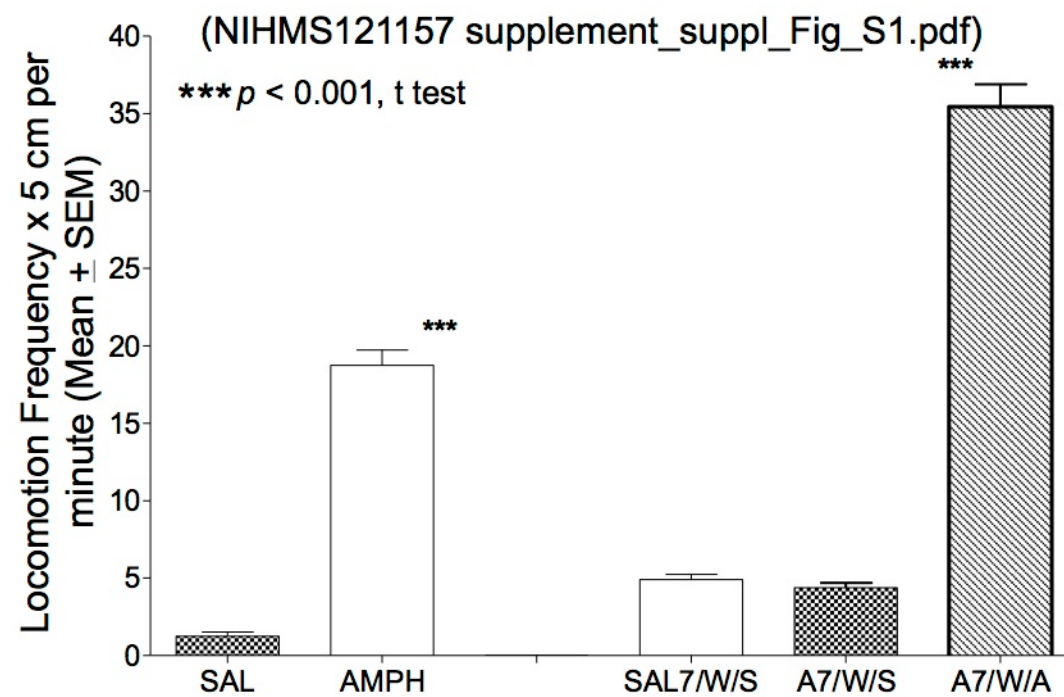

Supplement: Additional file 7: — We conducted a gross comparison of the total locomotor activities, and compared changes in locomotion between AMPH-treated and saline-treated animals in various exposure paradigms (SAL vs. AMPH groups in acute exposure, SAL7/W/S vs. A7/W/A and SAL7/W/S vs A7/W/S [placebo] in chronic exposure groups). Using one-way ANOVA followed by Newman-Keuls Multiple Comparison test, we found that AMPH induced a significant main effect (p < 0.001), with an exception between the SAL7/W/S and A7/W/S groups (p > 0.05). The average rate of locomotion (in meters per hour) was 57 + 6 and 105 + 6 for A1 and A7/W/A, respectively. (PDF 107 kb) [file 12929_2016_294_MOESM7_ESM.pdf]
