# Supplementary material for: Epigenetics of amphetamine-induced sensitization: HDAC5 expression and microRNA in neural remodeling
Source: J Biomed Sci. 2016 Dec 8;23:90. doi: 10.1186/s12929-016-0294-8 (PMC5146867; doi:10.1186/s12929-016-0294-8)

Acute paradigm, Nac (1657) Anti-HDAC5 antibody (ab1439)

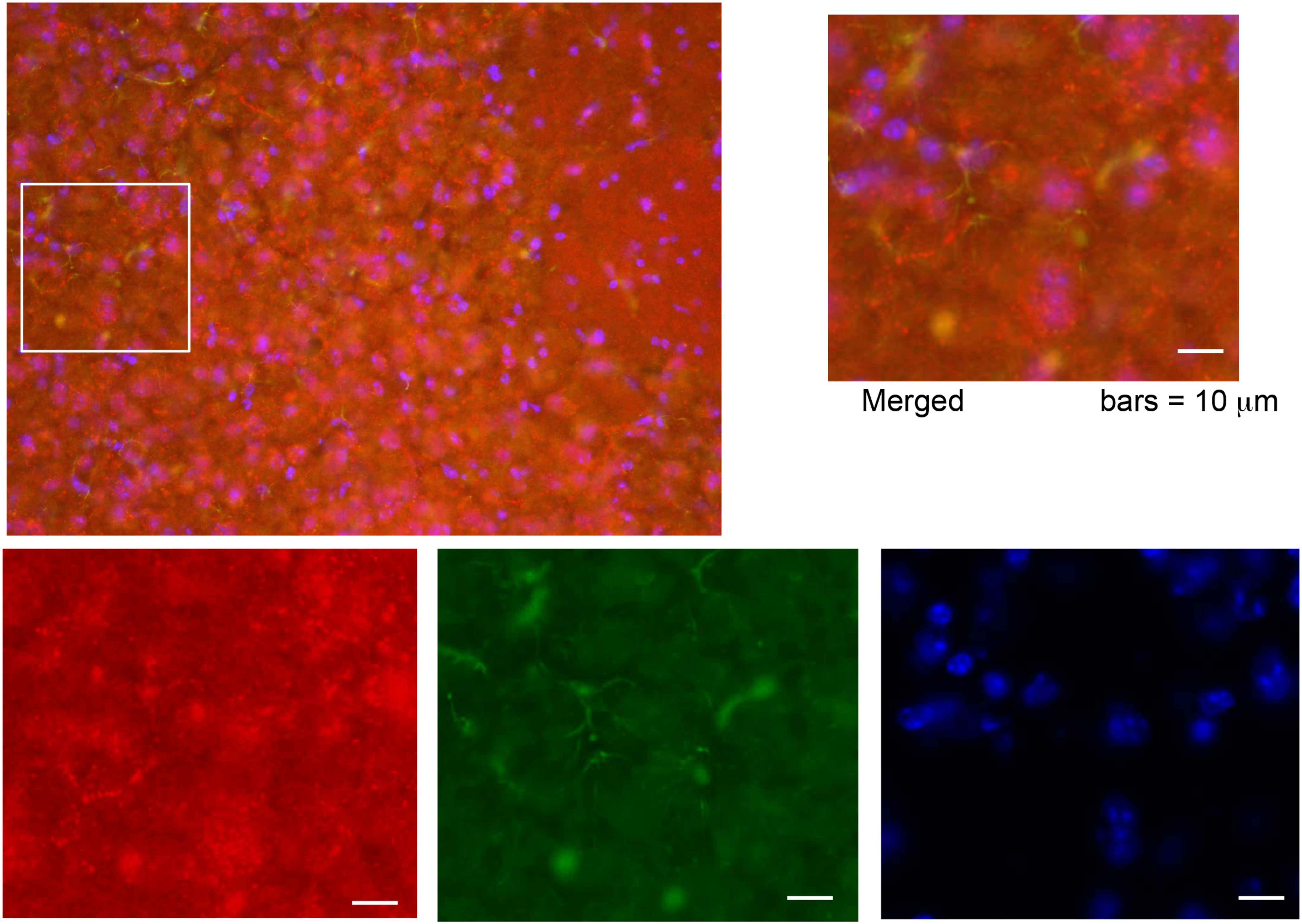

Merged bars = 10 μm

Acute paradigm, Nac (1657)

Anti-HDAC5 (phospho S259) antibody ab192339)

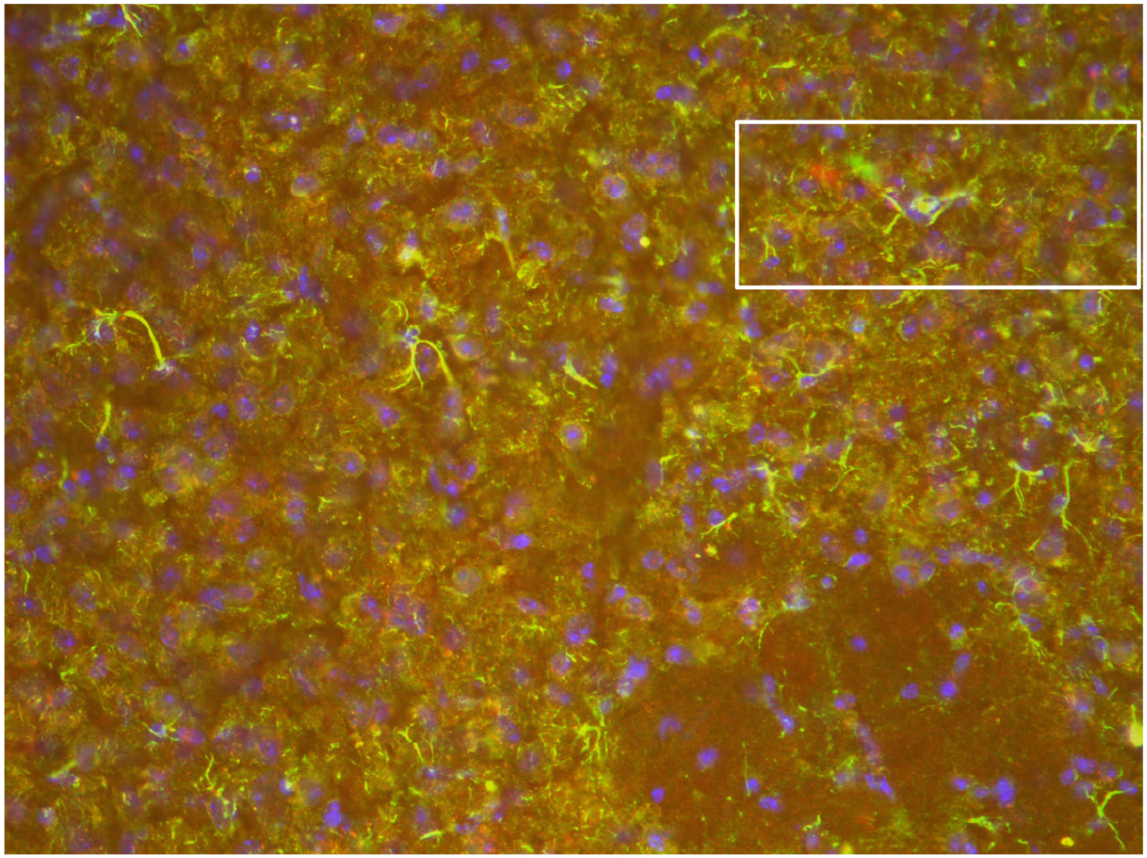

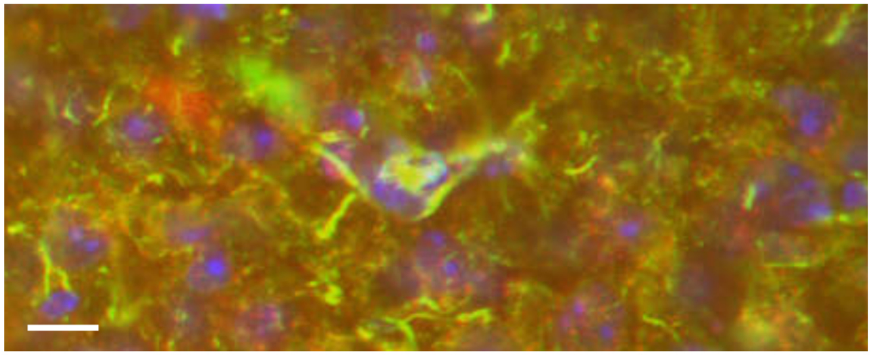

merged

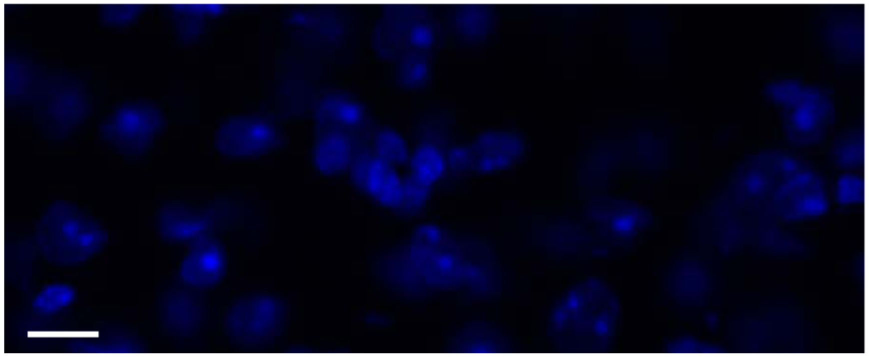

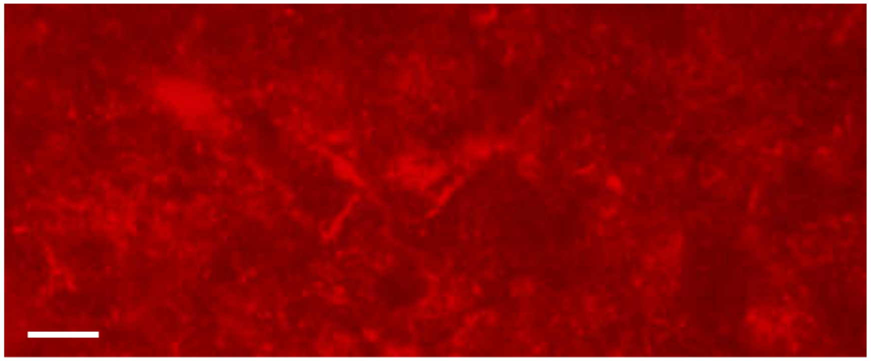

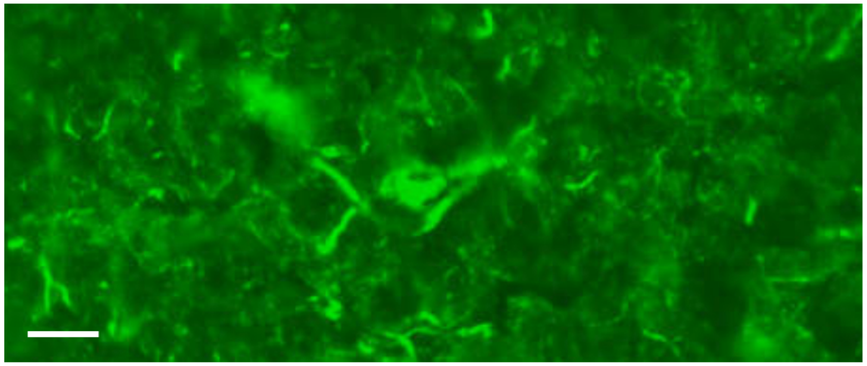

Supplement: Additional file 3: — Expression of HDAC5 antigens in mice of acute amphetamine exposure groups. We compared total (file 2) or phosphorylated (file 3) HDAC5 in the NAc. (PDF 3461 kb) [file 12929_2016_294_MOESM3_ESM.pdf]
