# Supplementary material for: Epigenetics of amphetamine-induced sensitization: HDAC5 expression and microRNA in neural remodeling
Source: J Biomed Sci. 2016 Dec 8;23:90. doi: 10.1186/s12929-016-0294-8 (PMC5146867; doi:10.1186/s12929-016-0294-8)

Chronic paradigm, Nac (1709) Anti-HDAC5 antibody (ab1439)

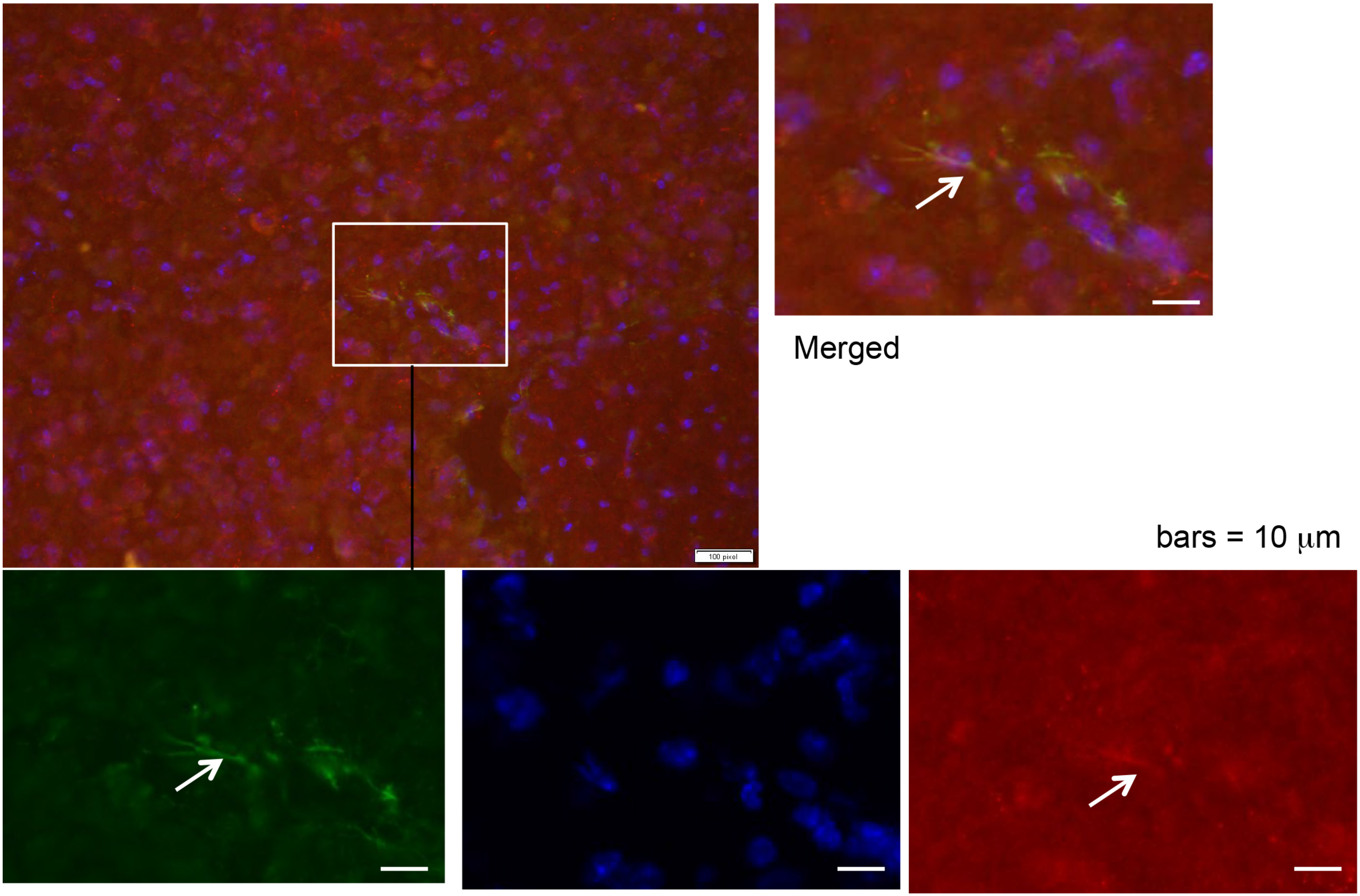

Chronic paradigm, Nac (1709)

Anti-HDAC5 (phospho S259) antibody ab192339)

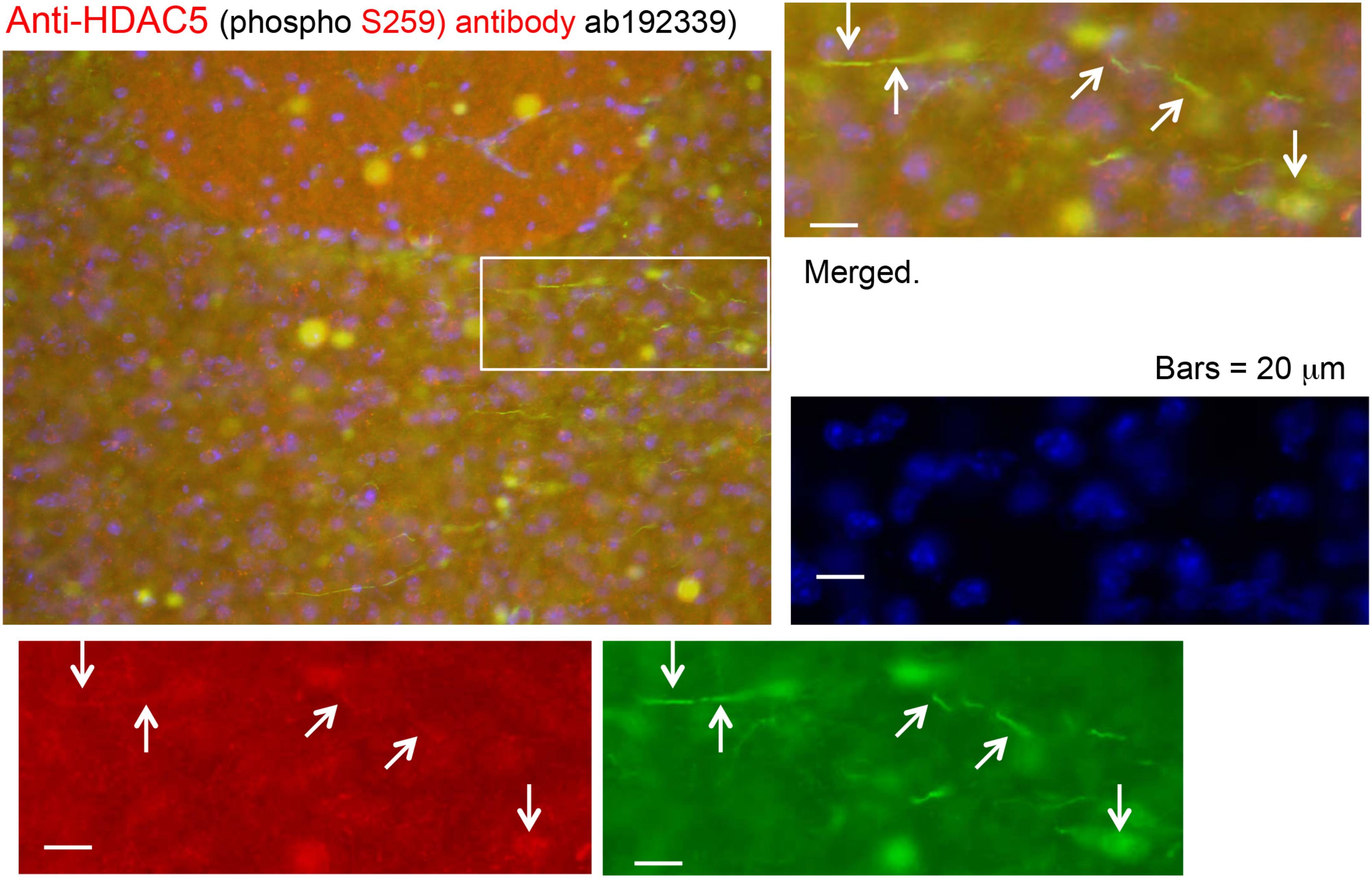

Supplement: Additional file 4: — Expression of HDAC5 antigens in mice in the chronic amphetamine exposure groups. We compared total (file 4) or phosphorylated (file 5) HDAC5 in the NAc. (PDF 2600 kb) [file 12929_2016_294_MOESM4_ESM.pdf]
