# Supplementary material for: Epigenetics of amphetamine-induced sensitization: HDAC5 expression and microRNA in neural remodeling
Source: J Biomed Sci. 2016 Dec 8;23:90. doi: 10.1186/s12929-016-0294-8 (PMC5146867; doi:10.1186/s12929-016-0294-8)
Supplement: Additional file 5: — We observed electron dense nanoparticles (EDN) in mice with (D, E, G & H) or without (A-C) SPION-sODN at the optimal dose (0.04 mg/kg, intracerebroventricular [icv] injection); there is no preferential accumulation of EDNs in mice with optimal dose of SPION-sODN. Accumulation of EDNs are observed in mice with 3X optimal dose (0.12 mg/kg, intracerebroventricular injection, not shown) or 8 optimal doses at one injection every week (4 mg/kg, intraperitoneal [i.p] injection, F). We attempted to identify SPION-sODN of 30 nm (dia) in Fig S4E; TEM stains masked our ability to identify SPION-sODN as EDN at ~30 nm (dia). (PDF 5888 kb) [file 12929_2016_294_MOESM5_ESM.pdf]

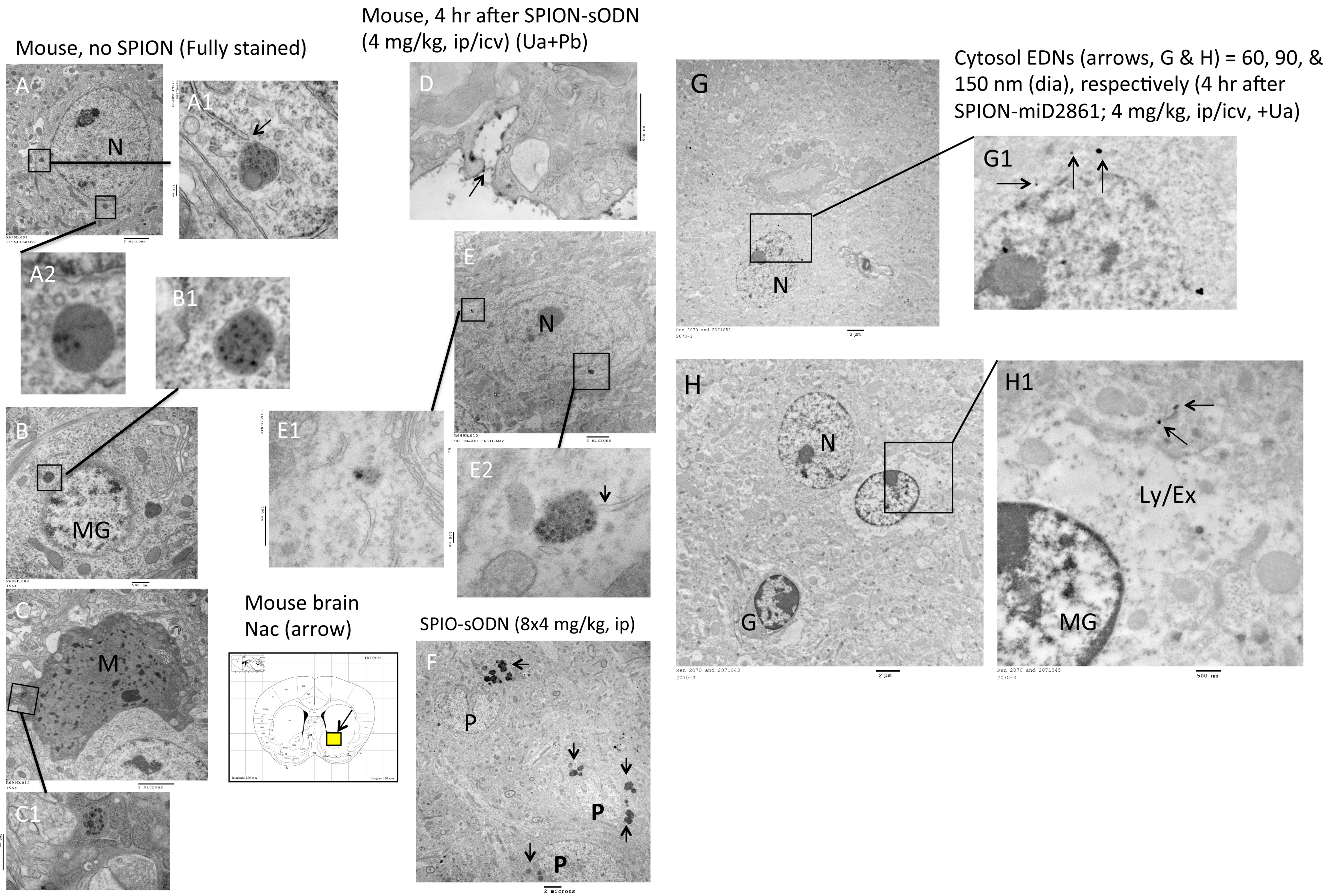

Mouse, no SPION (Fully stained)
A
N
A1
A2
B1
B
MG
C
M
C1
2 microns
500 nm
Mouse, 4 hr after SPION-sODN
(4 mg/kg, ip/icv) (Ua+Pb)
D
E
N
E1
E2
Mouse brain
Nac (arrow)
SPIO-sODN (8x4 mg/kg, ip)
F
P
P
P
2 microns
Cytosol EDNs (arrows, G & H) = 60, 90, &
150 nm (dia), respectively (4 hr after
SPION-miD2861; 4 mg/kg, ip/icv, +Ua)
G
N
G1
H
N
G
H1
Ly/Ex
MG
2 µm
500 nm
