# Supplementary material for: Epigenetics of amphetamine-induced sensitization: HDAC5 expression and microRNA in neural remodeling
Source: J Biomed Sci. 2016 Dec 8;23:90. doi: 10.1186/s12929-016-0294-8 (PMC5146867; doi:10.1186/s12929-016-0294-8)
Supplement: Additional file 6: — We observed several nuclear EDNs with a uniform diameter of 30 nm (A1, B1 & B2, arrowheads); three of these EDNs appeared on the membrane in tandem near the rough ER (A1). Only EDN larger than 60 nm appeared to be in the cytoplasm (B & B1, arrows). These unstained samples had reduced background noise, and we found EDNs (arrows) in the cytoplasm and nuclei. Although EDNs were visible, we cannot identify Ly/Ex, but can identify MG and N from the outline of their nuclei. Bars (microns) = 500 (A, A1 & B). (PDF 2836 kb) [file 12929_2016_294_MOESM6_ESM.pdf]

Mouse, +SPION-sODN (4 mg/kg, ip/icv) (Unstained)

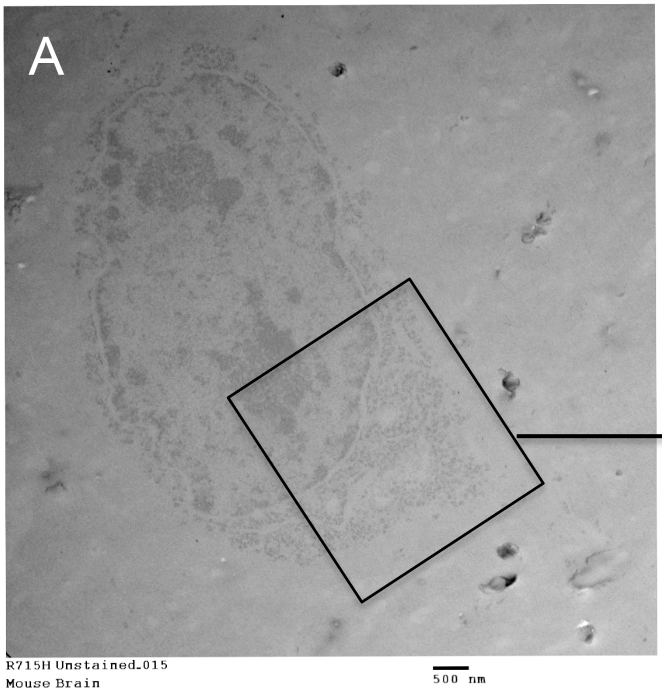

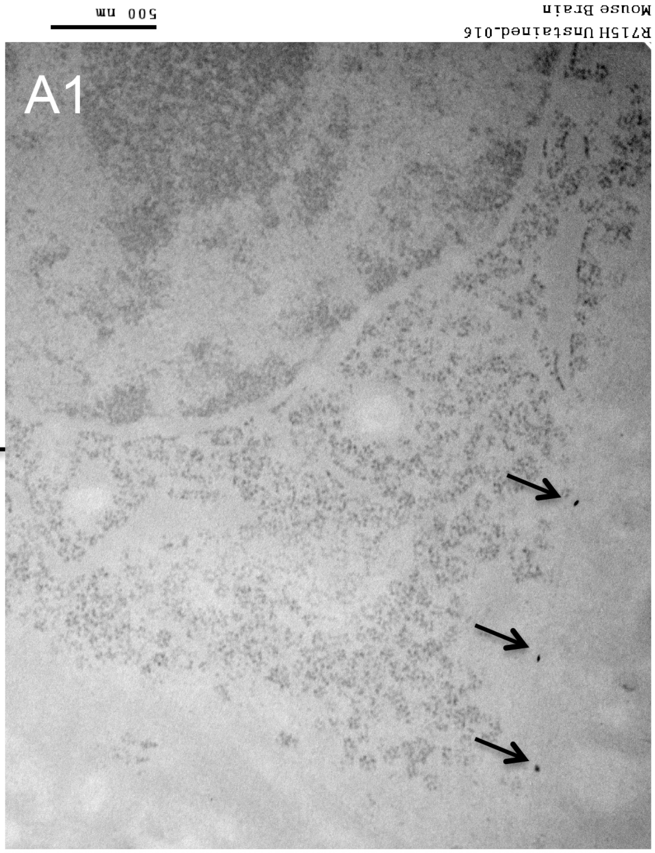

EDNs (arrows) ≤ 30 nm (dia)

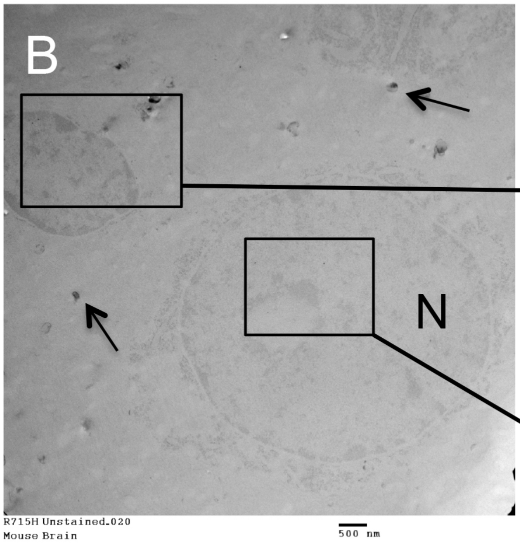

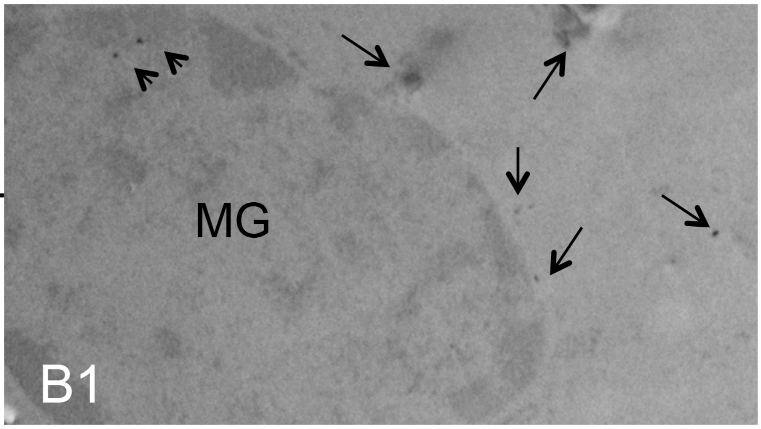

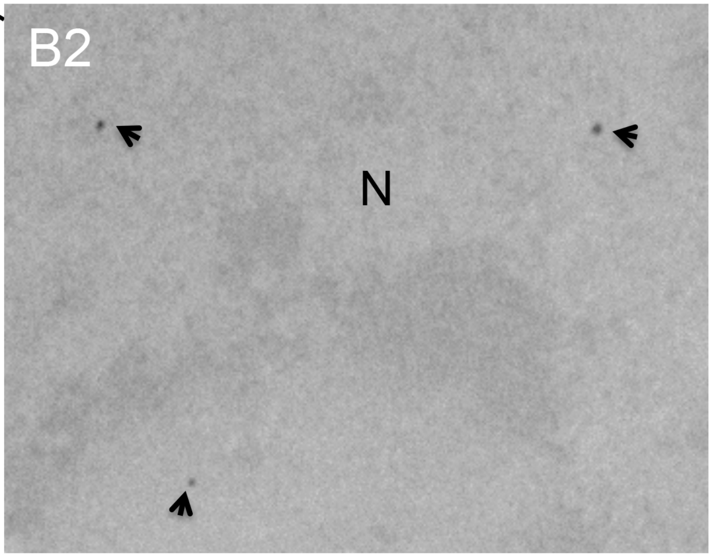

EDNs ≤ 30 (arrowhead) nm; ≥ 60 (arrows) nm (dia)
